# Managed rainforests support higher carbon density and sequestration in the Congo Basin

**DOI:** 10.1038/s41467-026-72399-4

**Published:** 2026-04-30

**Authors:** Le Bienfaiteur Sagang, Ricardo Dalagnol, Lee White, Stephanie George-Chacon, Samuel Favrichon, Shuang Li, Fabien Wagner, Zhihua Liu, Dafeng Zhang, Alfred Ngomanda, Vincent Medjibe, Bonaventure Sonké, Nicolas Barbier, Elsa M. Ordway, Sassan Saatchi

**Affiliations:** 1 Institute of the Environment and Sustainability, University of California, Los Angeles, CA USA; 2 Ctrees, Pasadena, CA USA; 3 School of Natural Sciences, University of Stirling, Stirling, UK; 4 Institut de recherche en écologie tropicale (IRET), Libreville, Gabon; 5 Global Green Growth Institute (GGGI), Mexico City, Mexico; 6 Gamma Remote Sensing AG, Gümligen, Switzerland; 7 Centre National de la Recherche Scientifique et Technologique (CENAREST), Libreville, Gabon; 8 Independent Researcher, Libreville, Gabon; 9 Plant Systematics and Ecology Laboratory, Higher Teachers’ Training College, University of Yaoundé I, Yaoundé, Cameroon; 10 AMAP, Univ Montpellier, IRD, CIRAD, CNRS, INRAE, Montpellier, France; 11 Department of Ecology and Evolutionary Biology, University of California, Los Angeles, CA USA; 12 Jet Propulsion Laboratory, California Institute of Technology, Pasadena, CA USA

**Keywords:** Ecosystem services, Environmental impact

## Abstract

Land-use is a key driver of forest loss and aboveground live carbon (AGC) emissions in the Congo Basin (CB) rainforest. Here we evaluate the influence of land-use disturbances on AGC stocks and fluxes by developing an AGC density map for the year 2020 and integrating it with high-resolution forest cover change data spanning 30 years (1990-2020) to quantify carbon emissions and removals. Logged forests show 8% (5%–10%) less AGC compared to old growth, while slash-and-burn and unmanaged degradations display up to 50% differences. Unmanaged areas account for 54% of the region’s AGC storage. Old growth dominates the total AGC removals (84%) with the region functioning as a net AGC sink at -37.5 ± 4.8 TgCyr^1^, driven by logging concessions (-21.3 ± 2.4 TgCyr^-1^) and protected areas (-15.7 ± 2.2 TgCyr^-1^), while unmanaged areas remained nearly neutral. These findings emphasize the role of sustainable forests management to enhance carbon retention in the region.

## Abstract (web summary)

Sustainable logging and improved forest management in the Congo Basin sustain livelihoods and boost carbon retention. Yet half of regional carbon storage remains vulnerable to unmanaged conversion, threatening climate mitigation and biodiversity.

## Introduction

The Congo Basin rainforest, the second-largest expanse of tropical forest globally, is home to an estimated 7000 endemic plant species^1^ as well as iconic animal biodiversity^2^ including bonobos, chimpanzees, gorillas, forest elephants, and okapi, and sustains the livelihoods of millions of people^3^. It is also the main driver of the world’s terrestrial carbon sink and harbors the largest tropical peatlands^4–7^. Combined pressures from climate change, subsistence slash-and-burn agriculture and increasing resource extraction resulting from human demand locally and globally^7–12^ threaten the integrity of this ecosystem with significant consequences for the Congo Basin’s carbon cycle, climate regulation across the basin and wider afield, and biodiversity^7,13–16^.

Long-term land-use management that safeguards forest resources and biodiversity while promoting sustainable income revenues is essential to successfully integrate the Congo Basin rainforest into global change adaptation and mitigation efforts^17^. Tropical forest live carbon stock and fluxes vary widely across gradients of functional composition, environmental drivers and disturbance regime^18^. However, the region remains understudied^19,20^, and the impacts of the main land-use activities, such as smallholder farming, logging, and associated canopy cover disturbances^12,21,22^, on forest structure and dynamics remain uncertain or underestimated^23^. This hinders our ability to assess how forest carbon stocks and fluxes vary across managed (logging concessions, protected areas) and unmanaged forests, predict their response to global changes and ultimately inform decision-making to protect and govern the Congo Basin rainforest in ways that also support economic growth. Traditional approaches used to characterize forest carbon allocation relied on sparse field inventory plot data that were often geographically biased and failed to adequately capture the variability of carbon density across gradients of forest functional traits and land-use intensity^7,24–27^. Airborne and spaceborne Light Detection and Ranging (LiDAR) technologies offer an opportunity to sample across functional and structural gradients at climate mitigation policy-relevant scales^28–31^, and interpolate forest measurements using wall-to-wall satellite products and machine learning (ML) techniques^32–34^. Furthermore, the release of high-spatial resolution (4.77 m) data from Planet’s constellation through Norway’s International Climate and Forest Initiative^35^ from 2015-2024 offered unprecedented spatial and temporal details (monthly to bi-annual) enabling a fine-scale delineation of areas impacted by logging, slash-and-burn practices, and the expansion of minor unpaved roads across the tropics as mapped by a deep learning method and data made available for visualization in the REDD + AI platform^36,37^. In addition, the democratization of nearly four decades of optical satellite image archives from Landsat^38^ allows for consistent, long-term characterization of changes in tropical moist forest (TMF) cover and the tracking of TMF gains and losses^21,39^ at moderate-spatial resolution (30 m), making it ideal for historical trend analysis^40,41^.

In this work we generate a wall-to-wall map of the aboveground carbon (AGC) density across the Congo Basin rainforest for circa 2020 at 100 m resolution by applying stratified ML models calibrated with extensive airborne LiDAR-derived AGC samples. This approach integrates canopy height metrics from the Global Ecosystem Dynamics Investigation (GEDI^28^) alongside optical and radar satellite predictors. We analyze the AGC distribution across the main forest types (central and coastal evergreen, semi-deciduous, peatlands and mangroves^42,43^), and land-use management regimes, including certified and uncertified logging concessions, protected and unprotected areas. We evaluate the influence of managed disturbances on AGC by combining our AGC map with the disturbance dataset from REDD + AI and comparing the AGC differences between old growth forests and those recently impacted by logging, slash-and-burn, and other forms of unmanaged disturbances. We combine the 30-year TMF cover change dataset (1990–2020) with our AGC map to quantify AGC recovery following stand clearing and degradation across different land-uses and forest types, using a space-for-time approach that models AGC accumulation as a function of the years since the last disturbance^40,41^. We integrate the carbon gains and losses across old growth, recovering, and disturbed forests to assess the AGC budget of the Congo Basin rainforests from 1990 to 2020. The estimates on carbon stocks and stock changes attributed to emissions and removals are all accompanied by uncertainty estimates that follow standard approaches^44–47^.

## Results

### Distribution of the aboveground carbon density across the Congo Basin rainforest

**Fig. 1:**
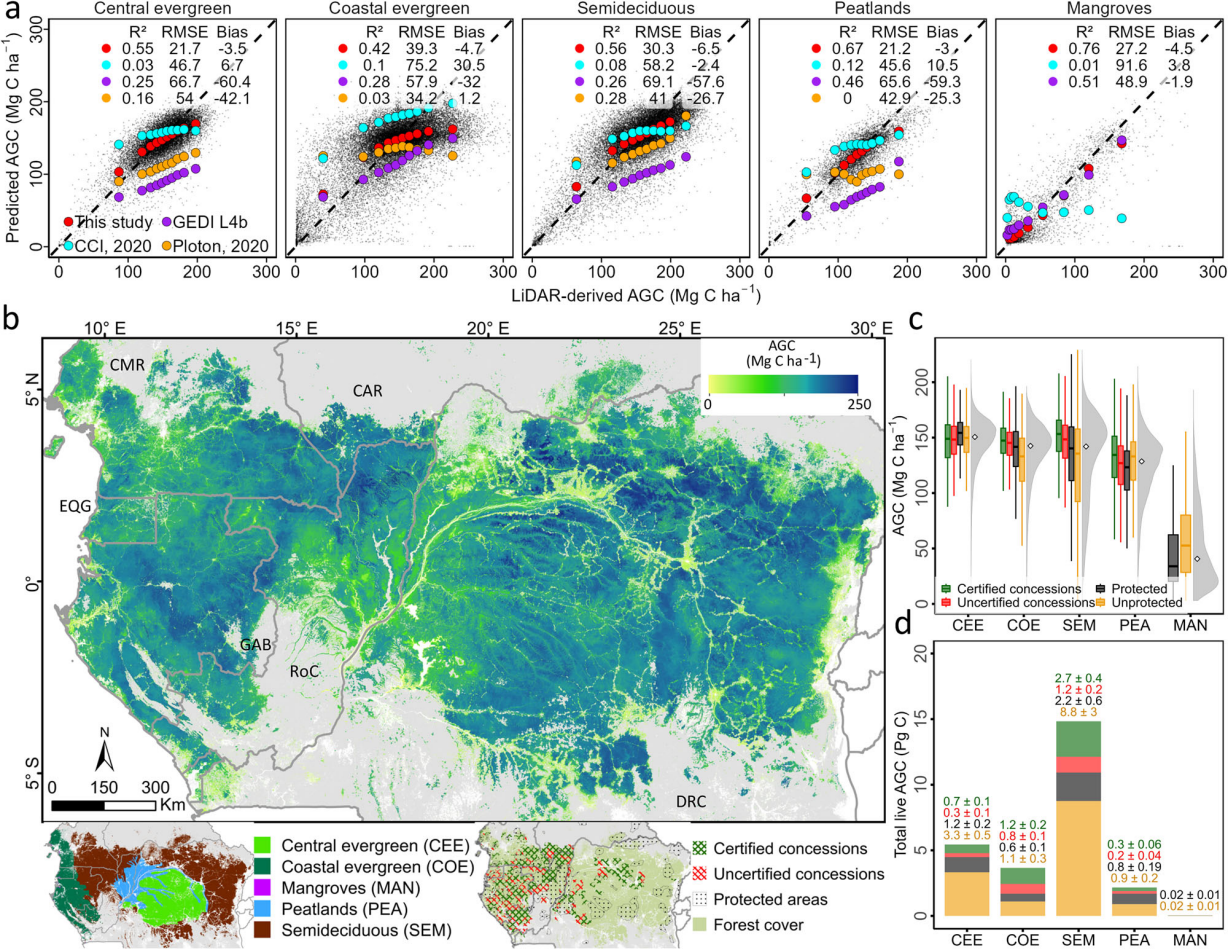
Aboveground carbon (AGC, Mg C ha^−1^) density and storage (Pg C) in the Congo Basin (CB) rainforest for the year circa 2020. **a** Performance of Gradient Boosted Regression Trees (GBRT) models for each forest type using a 10 spatial fold cross validation (see Materials and Methods for more details). The scatter plots compare airborne LiDAR-based AGC density with estimates from this study, Climate Change Initiative (CCI) of 2020, Global Ecosystem Dynamics Investigation (GEDI) L4b, and aggregated management inventory measurements^99^, with average estimates within 10 percentile intervals of the reference LiDAR-based AGC. **b** AGC density map with the inserts of forest types (^42,43^; bottom left) and land-uses (bottom right). CAR Central Africa Republic, CMR Cameroon; DRC Democratic Republic of Congo, EQG Equatorial Guinea, GAB Gabon, RoC Republic of Congo. **c** Distribution of AGC density across forest types (filled curves with their means as dots), and land use (boxplots). **d** Estimated total live AGC storage (Pg C) within forest types with the proportion allocated within each land-use. Area (Mha) of each vegetation type: Central evergreen (CEE) = 37.1, Coastal evergreen (COE) = 26.6, Mangroves (MAN) = 0.4, Peatlands (PEA) = 17.5, Semideciduous (SEM) = 113.2.

The AGC estimates derived from our Gradient Boosted Regression Trees (GBRT) models, trained on ~825,000 ha of reference LiDAR-derived AGC data outperformed existing global and regional products across forest types (Fig. 1, see the “Materials and Methods”), with averaged R² 0.6; RMSE = 28 Mg C ha^−1^ and bias = −4.5 (Fig. 1a).

The average AGC of 134.1 SD ± 35.1 Mg C ha^−1^ derived from our map was comparable to the estimate derived from nearly 100,000 ha of aggregated in situ forest management inventories in the region (134.3 ± 38.5 Mg C ha^−1^ ^48^; Figs. S4 & S5); but is 6% lower than estimates from 673 1-ha plot data (145.5 ± 51.5 Mg C ha^−1^; Fig. S5) and LiDAR-derived AGC data (143.6 ± 49.6 Mg C ha^−1^). We noticed a 7.5% overestimation (154.4 ± 38.7 Mg C ha^−1^; bias:-2.1 to 30.5 Mg C ha^−1^) with the 2020 biomass map from the Climate Change Initiative (CCI^49^), and 33.6% underestimation (95.3 ± 34 Mg C ha^−1^; bias: −60.4 to −1.9 Mg C ha^−1^) with GEDI L4B data^50^.

An ANOVA test revealed a significant difference in mean AGC across forest types (*p*-value < 2e-16), with a Tukey post hoc test showing that central evergreen forests had the highest AGC density (146.7 ± 22.2 Mg C ha^−1^, Fig. 1c) followed by coastal evergreen (137.5 ± 26.3 Mg C ha^−1^) and semideciduous forests (131.1 ± 39.4 Mg C ha^−1^), with lowest values found in mangroves (50.3 ± 34 Mg C ha^−1^). Managed forests within logging concessions were found to store 10% more AGC density on average (144 ± 25 Mg C ha^−1^) compared to other land-uses, except for central evergreen forests, where protected areas had the highest AGC density (150.4 ± 20.7 Mg C ha^−1^). Mangrove forests found within unprotected areas had higher AGC density (56.5 ± 33.1 Mg C ha^−1^) than protected mangroves (45.6 ± 33.9 Mg C ha^−1^). We estimated at 26.1 ± 3.2 Pg C the total live AGC stock in the Congo Basin rainforests in 2020, with 54.1% (14.1 ± 0.6 Pg C) found within unprotected forests and 46.1% (12.1 ± 0.9 Pg C) shared between logging concessions (7.3 ± 0.56 Pg C) and protected areas (4.7 ± 0.6 Pg C).

### Land-use drives aboveground carbon storage and fluxes

**Fig. 2:**
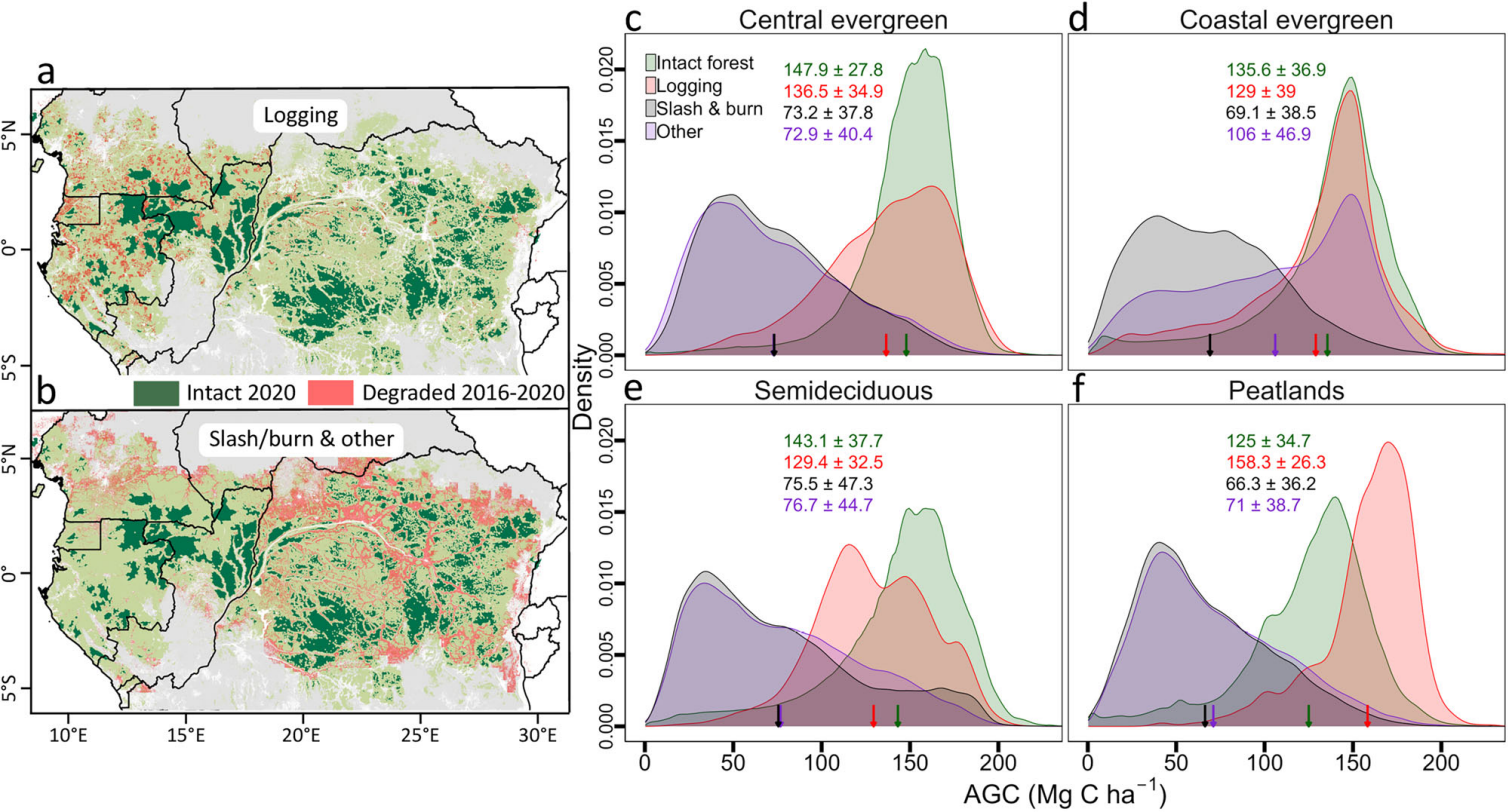
Influence of land-use disturbances on aboveground carbon (AGC, Mg C ha^−1^) density. **a**, **b** Spatial extent of forest disturbance and their drivers extracted and modified from the REDD + AI platform^37^ over the Congo Basin rainforest. These drivers were categorized into three types: logging activities (top left map), slash-and-burn disturbances, and other degradation, which includes forest roads and canopy openings outside logging concessions. The intact forest landscape (IFL) map of 2020^92^ was used to define the extent of old growth forests with no evident signs of recent disturbances as detected from satellite images. To reduce the effect of human disturbances on forest structure, the initial 1 km buffer around roads and settlements used as one of the criteria to define IFL was expanded to 4 km. **c**–**f** AGC distribution within disturbed and old growth forests for each forest type and land-use with their respective means and standard deviations. The AGC means for each land-use are indicated with arrows.

On average, AGC in selectively logged forests was 7.5% lower than in old growth forests (Fig. 2), with differences ranging from 4.9% in coastal evergreen forests (Fig. 2d) to 9.5% in semideciduous forests (Fig. 2e). Peatlands were an exception, where logged forests exhibited 26.6% higher AGC density (158.3 ± 26.3 Mg C ha^−1^) than old growth forests (125 ± 34.7 Mg C ha^−1^). Forests affected by unmanaged disturbances including slash-and-burn showed the lowest AGC density, reaching 50.1% less AGC than old growth forests.

**Fig. 3:**
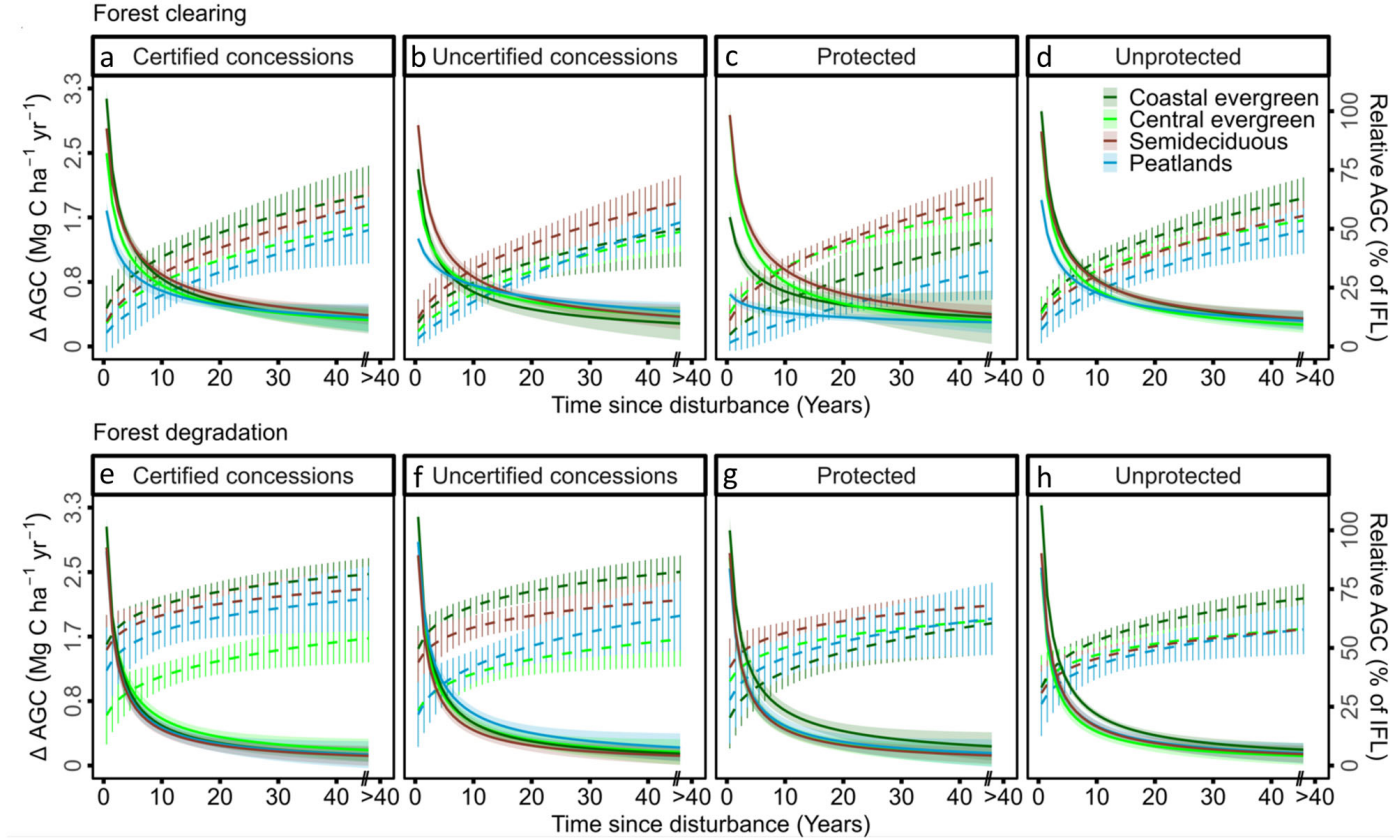
Influence of land-use disturbances on aboveground carbon (AGC) accumulation potential. AGC recovery following forest clearing (**a**–**d**) and degradation (**e**–**h**) across land-uses and forest types. Solid lines are the rates of AGC accumulation (∆AGC; Mg C ha^−1^ year^-1^; left y axis) with the associated 95% confidence interval and dotted lines are the estimated relative AGC density expressed as percentage of AGC of old growth forests found within intact forest landscapes (IFL, right y axis) with the associated 95% confidence interval.

Our results revealed distinctive patterns of AGC recovery over 30 years following forest clearing and degradation (Fig. 3). The average annual rates of AGC accumulation of recovering forests from clearing within the first 20 years after disturbance was 2.1 ± 0.2 Mg C ha^−1^yr^−1^ (Fig. 3a-d) with no significant differences across forest types and land-uses, except in protected areas (Table S1), where the highest rates were found in semideciduous forests (2.6 ± 0.2 Mg C ha^−1^yr^−1^) and the lowest rates in peatlands (1.6 ± 0.1 Mg C ha^−1^yr^−1^). Young ( ≤ 20 years) degraded forests displayed mean AGC accumulation of 1.7 ± 0.2 Mg C ha^−1^ yr^−1^ (Fig. 3e-h). Degradation within logging concessions maintained higher levels of AGC (above 50% of the mean AGC of old growth forests) within the first year after disturbance, except for central evergreen forests and peatlands. Older ( > 30 years) recovering forests displayed an average annual AGC accumulation of 0.6 ± 0.4 Mg C ha^−1^ yr^−1^, with the rate of recovery being 2.2 times higher following forest clearing (0.8 ± 0.3 Mg C ha^−1^ yr^−1^) compared to forest degradation (0.4 ± 0.3 Mg C ha^−1^ yr^−1^).

### Managed rainforests in the Congo Basin act as a net aboveground carbon sink

We applied our AGC accumulation models across all recovering ( < 30 years) and old growth forests (including undisturbed TMF cover in 2020 and those recovering beyond 30 years; Table S1) for each land-use and forest type in the Congo Basin rainforest, resulting in a total AGC sequestration of −3415.6 ± 273.2 Tg C from 1990 to 2020 (Table 1).

**Table 1:** Aboveground carbon (AGC) removals and emissions over the Congo Basin rainforest between 1990 and 2020

Land-use	AGC Removals			AGC Emissions		Total net change	Annual net change
	Old growth	Degraded	Regrowth	Clearing	Degradation		
Certified concessions	−589±64.8	−31.3±3.8	−0.6±0.1	75.5±5.7	120.6±9.8	−424.8±78	−14.2 ±2.6
Uncertified concessions	−284.6±30.3	−15.5 ±1.3	−0.3 ±0.04	36 ±2.7	50.2 ±4.1	−214.3 ±25.2	−7.1 ±0.84
Protected	−627.7 ±70	−35.2 ±5.7	−1.7 ±0.3	86.8 ±6.5	105.58 ±12.5	−472.2 ±57	−15.7 ±1.9
Unprotected	−1360.6 ±143.6	−454.8 ±54.7	−14.3 ±2	904.7 ±67.8	911.3 ±73.8	−13.8 ±3	−0.5 ±0.1
Total	−2861.9 ±314.8	−536.8 ±64.6	−16.9 ±2.4	1103 ±89.3	1187.7 ±94.1	−1125.1 ±144	−37.5 ±4.8

Removals (negative values, Tg C) include AGC accumulation within old growth, degraded and recovering forests from clearing, while emissions (positive values) include AGC losses from clearing and degradation of degraded and old growth forests. Total and annual net changes (loss – gain, Tg C year^−1^) are aggregates within each land-use over 30 years.

Old growth forests dominated the AGC removals across all land use (−2861.9 ± 314.8 Tg C, 83.8%) followed by degraded (−536.8 ± 64.6 Tg C; 15.7%) and forests recovering from clearing (−16.9 ± 2.4 Tg C; 0.7%). Integrating the areas of TMF cover loss with the associated AGC emissions from this study (Table S2) resulted in a total AGC emission in the Congo Basin rainforest of 2290.7 ± 183.4 Tg C. About 79% of the total emissions occurred within unprotected forests (1816 ± 136.2 Tg C), and 21% within managed areas (474.7 ± 25.82 Tg C). By integrating total AGC losses and gains from 1990 to 2020, we estimated a net annual AGC sink of −37.5 ± 4.8 Tg C yr^−^^1^ in the Congo Basin rainforest. Managed land-uses contributed 98.7% (−37 ± 4.3 Tg C yr^-1^) to these net removals with logging concessions and protected areas contributing 56.8% and 41.9%, respectively. In contrast, unprotected areas remained almost carbon neutral with −0.5 ± 0.1 Tg C yr^−1^ of net AGC removals.

**Fig. 4:**
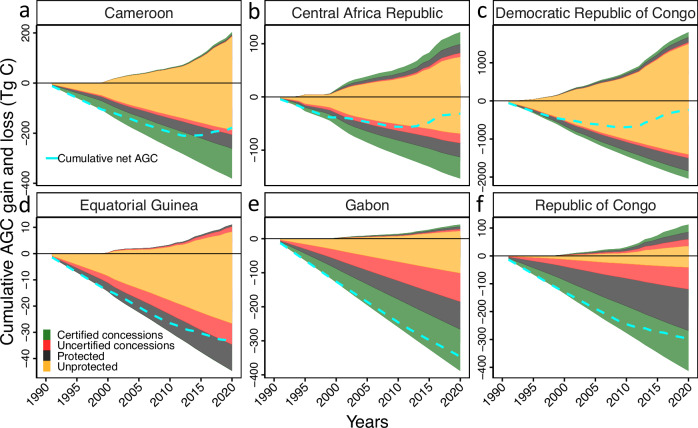
Aboveground carbon (AGC) budget for the Congo Basin rainforest over 30 years (1990–2020). Cumulative total annual AGC emissions (loss, positive values) and removals (gain, negative values) across land-uses for each of the Congo Basin countries; **a** Cameroon, **b** Central Africa Republic, **c** Democratic Republic of Congo, **d** Equatorial Guinea, **e** Gabon, **f** Republic of Congo; and their respective cumulative net balance (loss–gain, dotted line).

Equatorial Guinea (EQG), Gabon (GAB) and Republic of Congo (RC) showed a steady increase in the cumulative net annual AGC sink (losses−gains; dotted line in Fig. 4), although the rate decreased later in the time series for EQG and RC. Cameroon (CMR), the Central African Republic (CAR), and the Democratic Republic of Congo (DRC) showed a reduction in the net AGC sink after 2010. Gabon displayed the largest net AGC sink after 30 years (−11.6 ± 3.7 Tg C yr^−1^; Table S3), followed by the Republic of Congo (−10.1 ± 2.7 Tg C yr^−1^), with Central Africa Republic and Equatorial Guinea having the lowest net annual sinks (−1 ± 0.4 Tg C yr^−1^ and −1.2 ± 0.7 Tg C yr^−1^, respectively).

## Discussion

Our findings provide evidence that managed forests within logging concessions and protected areas sustain a higher aboveground carbon (AGC) density on average and act as the main carbon sink in the Congo Basin rainforests. These results are supported by our new and more accurate AGC map at 100 m spatial resolution, which enables a systematic assessment of the forest biomass allocation across forest types and land-uses.

The uncertainty in the AGC map-based inference for carbon density and changes across forest types and land uses remain small because of our innovative integration of satellite predictors with advanced geospatial modeling techniques. This includes: 1) employing locally trained machine learning (ML) algorithms within 20 km × 20 km grid cells to interpolate canopy height metrics from GEDI; and 2) leveraging the most extensive LiDAR-derived AGC samples available over the region (~825,000 ha; Table 3) to calibrate stratified ML algorithms for each forest type and predict AGC. The localized ML approach integrates the spatial variability in the forest structure and has been demonstrated to provide better performance in predicting canopy structure at regional^51,52^ and global^33^ scales. Upscaling AGC estimates from local to regional scales has been improved by integrating LiDAR-derived AGC samples^32,53^. The latter provides more comprehensive sampling and therefore a better characterization of the forest structure across the AGC gradient compared to limited field plot data. Global products either overestimated by 15% (CCI, 154.4 ± 38.7 Mg C ha^−1^) or underestimated by 28.9% (GEDI L4B, 95.3 ± 34 Mg C ha^−1^) compared to our regional mean AGC estimate (134.1 ± 35.1 Mg C ha^−1^). This underscores potential large uncertainties when relying on non-probabilistically sampled field inventory data or global maps to assess the contribution of the Congo Basin rainforest to global climate change mitigation efforts like the United Nations Framework Convention on Climate Change (UNFCCC), and highlights the need for locally calibrated, wall-to-wall products improved by intermediate LiDAR-derived AGC data.

Managed forests within logging concessions support 10% more AGC density compared to other land-uses, with certified logging concessions retaining notably higher AGC than that observed in protected areas. This aligns with a pattern already documented in previous studies in the region from field and remote sensing-based estimates^52,54,55^. The higher AGC density observed within logging concessions likely results from the combined effects of how they are established and operated. Unlike concessions, protected areas are established more recently and often on previously logged forests, or were given lower economic priority, making them more vulnerable to illegal land-use activities that negatively affect their AGC densities^56^. Logging concessions in Central Africa have a long history, beginning in colonial times and continuing through post-independence, initially dominated by European companies. More recently, Asian and North American companies have also established a presence. These concessions are typically located in areas rich with large, harvestable trees (minimum cutting diameter of 70 cm^57^), inherently supporting high AGC^27,58^. Within these areas, the selective logging practice removes only a small number of commercial stems per hectare (on average 0.7–2 trees ha^−1^), limiting carbon losses below 9%^59–63^. Our extensive data across the basin confirms this limited impact with logged forests showing on average 7.5% less AGC density (range: 4.9% - 9.5%) than undisturbed forests. Extended rotation cycles of at least 20 years between consecutive harvests, as implemented in Gabon^64,65^, further provide more time for the forest to recover between harvests, at higher rates of post-harvest carbon sequestration^24,55^. Finally, the closure of temporary roads within concessions^66^ restricts access to logged areas, reducing subsequent degradation from shifting agriculture, hunting, and unauthorized timber or fuelwood extraction. An exception is observed in central evergreen forests within the Democratic Republic of Congo (DRC), where the AGC density of uncertified logging concessions lacking a sustainable management plan (137 ± 30.2 Mg C ha^−1^ yr^−1^) is much closer to unmanaged forests (132.9 ± 38.6 Mg C ha^−1^ yr^−1^). This reflects the forestry laws in DRC, that allow a wide range of human activities within concessions, including the expansion of shifting agriculture^9,67^, unregulated timber harvesting for community needs^68,69^, human settlements^70^, and mining^8^. As a result, forest clearing and degradation inside concessions in DRC occur at rates two to three times higher than in other Congo Basin countries^66,71^.

Our estimates of annual AGC accumulation for young ( ≤ 20 years) forests recovering from clearing (2.1 ± 0.2 Mg C ha^−1^ yr^−1^) and degradation (1.7 ± 0.2 Mg C ha^−1^ yr^−1^) are similar but more conservative to previous estimates over Central Africa^40^ using a space for time approach (2.5 ± 2.4 Mg C ha^−1^ yr^−1^ and 2.0 ± 0.4 Mg C ha^−1^ yr^−1^). Our recovery rate for undisturbed forests ( > 30 years; 0.6 ± 0.4 Mg C ha^−1^ yr^−1^) falls within the lower range measured within field plots of old growth (0.5-0.8 Mg C ha^−1^ yr^−1,7^), indicating a potential lower carbon accumulation rate of old growth and undisturbed forests in the Congo Basin. Integrating belowground live carbon (BGC), using AGC-to-BGC conversion factors of 0.235 for *terra firme* forests^72^ and 0.39 for mangroves^73^, we estimate at 32.3 ± 3.2 Pg C the total live carbon storage (both aboveground and belowground) in the Congo Basin rainforest in circa 2020. This represents about 21% of the tropical moist forests total carbon stock (154 ± 1 Pg C^5^) and about seven times the annual global emissions from anthropogenic disturbances (4.6 ± 2.1 Pg C yr^−1,5^). This large carbon storage remains vulnerable to future land use change and illegal logging or clearing because about 54% of the carbon lies within unmanaged areas, and only 46% is found within managed land-uses and protection (18% in protected areas and 28% in logging concessions).

Managed forests are responsible for almost all (98.7%) of the net carbon sink of the Congo Basin rainforest, while unmanaged areas remain almost carbon neutral over the past 30 years. This highlights the need for strategic planning for climate change mitigation through nature-based solutions centered on the Congo Basin rainforest to effectively reduce Greenhouse Gas (GHG) emissions, enhance carbon sequestration and maximize the associated environmental and socioeconomic benefits to the community^74–76^. Where national circumstances permit, it is vital to prioritize the protection of old growth forests from deforestation and degradation^77^, as they account for a significant 83.8% to the total annual AGC gross removals estimated at 95.4 ± 7.6 Tg C yr^−1^. However, the Congo Basin rainforest exists within countries grappling with significant development challenges, heavily reliant on the economic benefits these forests provide^78,79^. The region is also expected to experience five-fold population growth by the end of this century, with expanded land-use pressure on the remaining old growth forests dominated by large- and small-scale agricultural development^10,12,80^. The Republic of Congo and Gabon offer a potential model for effective forest management; each boasting approximately 23 million hectares of forest cover as of 2020 (Fig. S7). Most of this forest area is managed, with 52.76% in concessions in the Republic of Congo and 56.26% in Gabon, along with 35.91% and 16.58% designated as protected areas, respectively. These concessions operate under legally binding management plans approved by the government, which adhere to standards comparable to those governing national forests in the United States and are classified as Type 6 protected areas by the International Union for Conservation of Nature (IUCN). Consequently, the net annual AGC sinks in these countries are over 1.3 times greater than the net sink reported in DRC, which recorded -7.7 Tg C per year. DRC, the largest forested country in the region with 115 million hectares of rainforest cover, has only 27.62% of its forests under management. Between 1990 and 2020, DRC experienced the largest reduction of its old growth forests with a decline of 32%, equivalent to 33.7 Mha (Figs. S7 & S8), primarily driven by forest cover loss in unmanaged areas (29.4 Mha). Gabon and the Republic of Congo also illustrate that effectively managed forests can promote positive biodiversity outcomes^81^. Notably, logging concessions in Gabon provide habitat for approximately 61,822 forest elephants, representing about 65% of the total national estimate (95,110^82^). This highlights the potential of sustainable forest management to address global challenges while serving as a foundation for national development, including the possibility of unlocking new nature-based financial opportunities through carbon markets, biodiversity credits, and climate funding^83^. However, financial mechanisms like Reducing Emissions from Deforestation and Forest Degradation (REDD + ) have yet to gain traction in the Congo Basin. Recently, the voluntary carbon market faced challenges amidst criticisms regarding result integrity and transparency, alongside shifts in methodologies^84^. Currently, Gabon is the only country in the region with REDD+ results registered on the UNFCCC Lima Hub^85^. Despite having validated CO_2_ emission reductions of 187 million tons from land-use changes, payments have been made for only 3.4 million tons.

Additional economic opportunities from the forest sector, such as removals from recovering forests, afforestation, improved management, and development of secondary or tertiary products from harvested forests can support overall conservation and sustainability of climate and biodiversity core benefits in the region. For example, Gabon enforced a ban on unprocessed timber exports in 2010. Investment in local timber processing, which increases the economic value and created jobs, triggered a threefold increase in forest-sector employment and a 322% rise in its contribution to national GDP by 2022^86^. Extending this model to other Congo Basin countries might improve the economic incentives to preserve standing forests and reduce the threats from future deforestation and degradation. Furthermore, abandoned logging roads often become hotspots for illegal activities such as poaching that greatly affect endangered species and threaten biodiversity worldwide^87^, therefore, safeguards to prevent this must be integrated into forest management and investment plans and environmental and social impact mitigation packages. Forest management in the Congo Basin has great potential to simultaneously mitigate climate change, safeguard forest resources and biodiversity, while enabling sustainable development through income generation. Yet achieving lasting impact will require increased investment in local timber processing as well as improved forest monitoring and governance.

## Method

### Forest types and land-uses

The forest composition map^42^ available over the Congo Basin at 10 km resolution was combined with the ecoregion boundaries^43^ to define three main forest types: Coastal evergreen (COE), Semideciduous (SEM) and Central evergreen (CEE) as described in Table 2.

**Table 2:** Criteria for determining forest types and the respective forest cover (Mha) in 2020 based on tropical moist forest (TMF) dataset

Réjou-Méchain et al. ^42^	Dinerstein et al. ^43^	This study	Area (Mha)
1-Atlantic highland evergreen	Congolian coastal forests; Cross-Sanaga-Bioko coastal forests	Coastal evergreen (COE)	26.609
2-Atlantic coastal evergreen			
3-Atlantic inland evergreen			
4-Margin semideciduous	Northern Congolian Forest-Savanna; Northwest Congolian lowland forests; Northeast Congolian lowland forests; Southern Congolian forest-savanna	Semi deciduous (SEM)	113.166
5-Evergreen semideciduous on sandstone			
6-Semideciduous			
8-Mixed evergreen			
9-Degraded semideciduous			
10-Semideciduous evergreen transition			
7-Central evergreen	Central Congolian lowland forests	Central evergreen (CEE)	37.118
--	Bunting et al. ^89^	Mangroves (MAN)	0.741
	ESA CCI^88^	Peatlands (PEA)	17.49

The COE class included all the Atlantic forests, which showed little taxonomic affinity from management inventories with the CEE class. We combined deciduous and semideciduous forests into one SEM class as they show functional convergence from the management inventory data^42^. The extent of terrestrial flooded forests, here referred to as peatland (PEA), was determined from the 2020 global land cover map from the European Space Agency and Copernicus Climate Change^88^ available at 300 m resolution. Finally, we used the global mangrove extent in 2020^89^ available at 25 m resolution to delineate mangroves (MAN). We derived our 2020 forest extent baseline from the 30 m Tropical Moist Forest (TMF) dataset^21^, resampled to 100 m resolution by including only pixels containing at least nine 30 m forested pixels.

Our baseline of forest cover at 100 m was used to resample all the different forest type maps using the nearest neighbor algorithm to fill gaps, resulting in a 100 m forest type map with five classes: CEE, COE, SEM, PEA, MAN. We categorized forest land-uses based on the type of management the forest was assigned for in 2020, including logging concessions, protected areas, and unprotected forests. We differentiated logging concessions with an officially validated sustainable forest management plan (Certified concessions) from those that either do not have an official management plan or have uncertain status (Uncertified concessions)^42^. Protected areas were extracted from the World Database of Protected Areas (WDPA)^90^. We excluded marine, hunting, and game-oriented areas. Unprotected forests were remaining forested areas that fell outside all the abovementioned management categories.

### Forest disturbance data

We used pantropical forest degradation dataset derived from deep learning (DL-DEGRAD^36^) and available for visualization on the REDD + AI platform^91^ to delineate the extent of areas impacted by logging practices, forest fire from slash-and-burn, and other types of degradation, including unpaved road openings. The DL-DEGRAD applies a U-Net model to detect combined tree loss and textural variations over very high spatial (4.77 m) and temporal (bi-annual to monthly) resolution Planet NICFI images from 2016 to 2020^35^. The multi-year/annual DL-DEGRAD dataset over the Congo Basin was consolidated into a single degradation raster, with adjacent pixels of the same driver merged into distinct polygons. To refine these polygons, we integrated the tree cover loss dataset from Global Forest Change (GFC^39^) and the Tropical Moist Forest (TMF^21^) to filter out the DL-DEGRAD polygons where no disturbance was detected by both former dataset between 2016 and 2020. We defined three categories of disturbances: 1) logging, corresponding to all logging detection happening within concessions which either follow certified management practices with reduced impact logging or uncertified practices with more damage to the forest^60^, 2) slash-and-burn, as all forest fire detection following forest clearing for small-scale agriculture, and 3) other disturbances, including forest roads and unmanaged canopy openings outside logging concessions. The intact forest landscape map (IFL^92^) was used to define the extent of stable old growth forests with no evident signs of recent disturbances as detected from satellite images. We limited the edge effect from the IFL map by expanding the initial 1 km buffer around roads and settlements used in the original IFL map to 4 km, since ~80% of degradation and deforestation in the Congo Basin occur within 4 km of road or settlement^12^.

### Mapping canopy height

Satellite data from the Harmonized Landsat Sentinel-2 (HLS^93^) and ALOS PALSAR^94^ available in 2020, were used as variables to interpolate canopy relative height (RH) measurements from GEDI^28^ into wall-to-wall canopy height maps. Optical inputs included the near-infrared (NIR), shortwave infrared (SWIR 1 & SWIR 2), and the Normalized Difference Vegetation Index (NDVI), while the horizontal (HH) and vertical (HV) polarizations were considered as predictors from radar. We retained the GEDI metrics RH50, RH75, and RH98 corresponding to the 50th, 75th, and 98th percentile of energy return height relative to the ground to capture the vertical structure of the different strata of the forest. The 25 m GEDI data available over the area between 2019 and 2020 were aggregated to a 100 m resolution using spatial averaging. We then set aside 20% of the 100 m GEDI pixels randomly selected for validation and the remaining 80% were subsequently incorporated into localized Gradient Boosted Regression Trees (GBRT^95^) models. GBRT models have been proven successful in predicting forest structural metrics with good performance^5,96^. These models (GBRT-RH) were trained and applied within 20 × 20 km tiles with 70% overlap, ensuring that a minimum of 500 GEDI pixels were available to train the model within the current tile and randomly sampling pixels from adjacent tiles if the condition was not met (see ref. ^52^ for more details). This resulted in three canopy height predictions (RH50, RH75, and RH98) for each tile for circa 2020 at 1-ha resolution. A wall-to-wall RH prediction over the Congo Basin (Fig. S1) was obtained by mosaicking estimates from each tile, considering the median value of overlapping pixels to minimize discontinuity effects between tiles^51^.

### Mapping aboveground live biomass

We gathered extensive airborne LiDAR-derived aboveground biomass (ALS-AGB; Figs. S4 & S5, Table 3) data available across the main forest types within the CB at 100 m^47,52,97,98^ to train GBRT-AGB models specific to each forest type. We excluded ALS-AGB pixels for which a canopy cover loss was recorded between the time of acquisition and 2020, resulting in ~825,000 pixels available for training at 1-ha (see Fig. S4a for the size of training sample within each forest type). The GBRT models to predict AGB (GBRT-AGB) included the HLS and radar predictors in addition to the predicted RH maps, and the Digital Elevation Model (DEM) from Copernicus. The performance of each GBRT-AGB model was assessed using a spatial cross validation approach. The dataset was split into 10 spatial folds created by clustering the coordinates of the pixels using the k-means algorithm (Fig. S4b). The model was trained 10 times and for each iteration, one cluster was set aside to validate the model while the remaining folders were used for training. Training data over PEA and MAN were split into 5 folds considering their smaller extent to keep a sufficient size of data for validation ( ≥ 500 pixels). We compared our results with 1) the AGB product from the European Space Agency (ESA) through the Climate Change Initiative (CCI) version 4^49^; 2) the AGB product from GEDI L4B^50^; 3) the aggregated AGB map from forest management inventory data in the Congo Basin (CoFor-AGB^99^) and 4) the AGB measurements from 673 1-ha plots from the cafriplot network and the national forest inventory (NFI) in Gabon. We used a factor of 0.456 to convert AGB to carbon density (AGC), and an ANOVA test was applied to compare the mean AGC density across management and forest types. The total live AGC stock was obtained by multiplying the AGC density with the total forest area in 2020, as determined by TMF forest cover.

**Table 3:** LiDAR-based reference aboveground carbon maps over the Congo Basin used in this study

Dataset	Country	Acquisition Year	Area (ha)	AGC (Mg C ha^-1^)	Relative RMSE (%)	R²
Xu et al. ^47^	Democratic Republic of Congo	2016	582,000	115.32 ± 62.51	20.3	0.82
Rodda et al. ^97^	Cameroon, Gabon	2012–2022	44,750	77.78 ± 58.29	15.2	0.89
Armston et al. ^98^	Gabon	2016	320,018	102.14 ± 39.43	25.72	0.86

### Quantifying carbon emissions and recovery

We used the TMF forest cover change dataset to track the trajectory of forest carbon recovery from when the forest was at its lowest AGC following stand clearing (defined as deforestation in the dataset) and degradation. This TMF dataset is a Landsat based monitoring of canopy gain and loss since 1984, at a spatial scale of 30 m (Figure S6). The TMF dataset can be used to estimate the time since the last disturbance event, which is considered a good proxy of the age of the recovering forest. We combined the TMF-derived forest age map with our predicted 2020 AGC map to determine the AGC with increasing age using a space for time approach as described by ref.^40^. We grouped spatially contiguous recovering pixels of the same year of disturbance into clusters and filtered out clusters with less than 9 pixels (0.81 ha) to align with the 100 m spatial scale of the AGC map. Furthermore, we removed plantation areas from TMF, recovering forests using the pantropical oil palm cover available for the year 2019^100^ and the 2020 land cover map for ESA^88^. This resulted in about 80 thousand clusters from which we extracted individual values of median AGC. We used the Chapman-Richard model for growth^101^ to model the AGC accumulation with increasing age of regrowing forest for each of the land-use within each forest type:

1 $${Y}_{t}=A{\left(1-{e}^{-{kt}}\right)}^{c}\pm {{{\rm{\varepsilon }}}}{;A},\,k \, {{{\rm{and}}}} \, {{{\rm{c}}}} > 0$$

Where ${Y}_{t}$ refers to the AGC at year (*t*); $A$ is the AGC asymptote or the AGC of the old growth forest; $k$ is a growth-rate coefficient of $Y$ as a function of age; $c$ is a coefficient that determines the shape of the growth curve; and *ɛ* is an error term.

We quantified the median AGC within different ages of recovering forests. We considered the 25^th^ quantile of AGC for 1-year old recovering forest from clearing to ensure that the fit begins near 0 at that age. This correction was not necessary for degraded forests as degradation still maintains some level of AGC after disturbance^40^. After a given number of years, the AGC of recovering forests would return to amounts equivalent to undisturbed old-growth forests which will constitute the precalculated asymptote *“A”* in our equation. For each forest type, we considered the 95th quantile of AGC found within old growth forests in 2020 (IFL^92^) as the respective *“A”* term in the equation. The equation was implemented using the ‘nls’ function in R statistical software^102^. The difference in estimated carbon between consecutive years was considered as the yearly carbon accumulation (∆ AGC, Mg C ha^−1^ year^−1^).

### Quantifying forest carbon gains and losses

To quantify the carbon gains of the region, we multiplied our modelled rates of annual AGC accumulation with the respective areas of forests recovering from clearing and degradation for each forest type and land use. We used the mean rate of recovering forests beyond 30 years (0.59 ± 0.42 Mg C ha^−1^ yr^−1^; Table S1) as the removal factor for old growth forests which include undisturbed TMF cover and old growth forests ( > 30 years). This estimated carbon removal rate of old growth forests falls within the range measured from field plots in the region (0.53 - 0.79 Mg C ha^–1^ yr^−1,7^. We added all AGC gains from the different pools to obtain the total AGC gain (Tg C). Total AGC losses were derived by combining emissions from forest clearing and degradation. For clearing, we used the TMF deforestation dataset to identify the year of pixel loss from old growth and degraded forests and multiplied the total cleared area between 1990 and 2020 by their respective median AGC, assuming complete carbon loss. For degradation, we estimated losses as the difference between AGC in old growth forests and the modelled AGC during the first year following the degradation event^40^ for each forest type and land use. These differences served as emission factors for degraded forests and were multiplied by the degraded forest area in 2020 to quantify total AGC losses from degradation. The net carbon budget for each land-use and forest type was then determined by calculating the difference between total carbon gains and losses from 1990 to 2020.

### Uncertainty analysis

We independently computed pixel-level uncertainties associated with RH and AGC predictions (GBRT-RH and GBRT-AGC). For GBRT-RH, we used the 20% validation dataset for each of the three RH metrics (RH50, RH75, and RH98) to calculate the average root mean squared error (RMSE) of the RH predictions within successive bins of 10% percentiles and fitted a function to describe how the absolute and relative errors propagate along a gradient of RH predictions (Fig. S2). To assess the uncertainties associated with AGC estimates, we assumed the RH metrics as predictor layers without any uncertainty and consider the variance of AGC density inference at the regional level to include both sampling and the residual variance. To assess the uncertainty of regional mean, we followed estimates of mean squared error (MSE) for the prediction of a population including the two errors:

2 $${\bar{\sigma }}^{2}={\bar{\sigma }}_{f}^{2}+{\bar{\sigma }}_{R}^{2}$$

Where ${\bar{\sigma }}^{2}$ is the total estimated uncertainty, ${\bar{\sigma }}_{f}^{2}$ is the sampling uncertainty and ${\bar{\sigma }}_{R}^{2}$ is the residual variance.

To assess the sampling variance of each pixel, we used a bootstrap approach, splitting the LiDAR-derived AGC data into 10-spatial folds and randomly assigning 80% of pixels into each fold while repeating the procedure 100 times. Over each iteration, 9 folds were considered for model training, while the left aside fold was used for model validation. The variance for the population mean was approximated as:

3 $${\bar{\sigma }}_{S}^{2}=\frac{1}{{n}_{{boot}}-1}{\sum }_{m=1}^{{n}_{{boot}}}({\mu }^{m}-{\bar{\mu }}_{{boot}})$$

Where ${n}_{{boot}}$ is the number of bootstrap iterations, ${\mu }^{m}$ is the mean of the population estimation for each iteration, and ${\bar{\mu }}_{{boot}}$ is the mean of population after ${n}_{{boot}}$ iterations.

The residual variance on the other hand includes two terms; one that provides the residual error between the pixel prediction and the mean AGC that is often negligible when calculated over large areas because it reduces by 1/N2, where N is the number of pixels covering the area. The second term includes the spatial correlation that is often difficult to calculate and requires semi-variogram analysis^44^ and contributes substantially for small areas but becomes also negligible for large areas whose dimensions is considerably greater than the spatial correlation length^45^.

4 $${\bar{\sigma }}_{R}^{2}=\frac{1}{{N}^{2}}\left({\sum }_{i=1}^{N}{\sigma }_{\varepsilon,i}^{2}+{\sum }_{i=1}^{N}{\sum }_{j\left(j\ne i\right)}^{N}{\rho }_{{ij}}{\sigma }_{\varepsilon,i}{\sigma }_{\epsilon,j}\right)$$

where ${\rho }_{{ij}}$ is the correlation coefficient between pixels $i$ and $j$, and it can be approximated from the variogram analysis under the assumption that spatial autocorrelation only changes with distance between the two pixels.

The uncertainty associated with the AGC accumulation models was quantified using a Monte Carlo simulation approach. We computed the median value of 50 clusters randomly selected from each chronosequence defined based on the time since the last disturbance. We repeated the procedure 100 times, and for each iteration, we used the Chapman-Richard model for growth^101^ to model the AGC accumulation with increasing age of regrowing forest for each of the land-use within each forest type. We quantified the rate of carbon accumulation for each iteration and determined a 95% confidence interval.

### Reporting summary

Further information on research design is available in the Nature Portfolio Reporting Summary linked to this article.

## Supplementary information

Supplementary information

Reporting Summary

Transparent Peer Review file

## Data Availability

All the satellite data are freely available from the Google Earth Engine repositories at https://developers.google.com/earth-engine/datasets/catalog. The Relative Height data from GEDI at footprint-level were downloaded from the GEDI02_A height and elevation product^28^, available at LPDAAC: 10.5067/GEDI/GEDI02_A.002. The Biomass dataset from GEDI’s was downloaded from the GEDI04_A product^50^ available at LPDAAC: 10.3334/ORNLDAAC/2056. The floristic maps from Réjou-Méchain et al. ^42^ and Dinersteirn et al. ^43^ are available at 10.18167/DVN1/UCNCA7 and https://hub.arcgis.com/datasets/esri::resolve-ecoregions-and-biomes/about. The mangrove dataset from Bunting et al. ^89^ is available at https://zenodo.org/records/6894273. The Intact Forest Landscape dataset for 2020^92^ can be downloaded at https://intactforests.org/data.ifl.html. Forest concessions in 2020 were downloaded at https://data.globalforestwatch.org/documents/gfw::managed-forest-concessions-downloadable/about. The extent of protected Areas in 2020^90^ was downloaded at www.protectedplanet.net. The tree cover change data from TMF^21^ and GFC^39^ can be accessed at https://forobs.jrc.ec.europa.eu/TMF/data.php#gee and https://earthenginepartners.appspot.com/science-2013-global-forest/download_v1.7.html. The forest degradation dataset displayed on the REDD + AI platform^91^ is subject to licensing agreement and available for research purposes upon request via CTrees.org/contact. The ESA biomass^49^ and vegetation types^88^ data are available at https://climate.esa.int/en/data/#/dashboard. The AGB map from management inventories^99^ is available at 10.6084/m9.figshare.11865450. The LIDAR-derived AGB data^97,98^ are available at 10.23708/H2MHXF, https://daac.ornl.gov/cgi-bin/dsviewer.pl?ds_id=1775 and Xu et al. ^47^. The AGB data from National forest 1-ha field plots are available from the cafriplot network; the Centre National de la Recherche Scientifique et Technologique (CENAREST) of Gabon (alfred.ngomanda@cenarest-gabon.org) and Xu et al. ^47^ (92 plots over DRC). The datasets required to reproduce our results have been deposited on Zenodo^103^ and available via this link: 10.5281/zenodo.18930528.

## Code availability

Satellite and spaceborne data processing was implemented in the GEE cloud computing platform following the guidelines available on GEE repository^104^. The rest of the processing was implemented in R statistical software (version 4.3.1), and the code to generate the results is available on Zenodo^103^ and available via this link: 10.5281/zenodo.18930528.
