# Supplementary material for: Managed rainforests support higher carbon density and sequestration in the Congo Basin
Source: Nat Commun. 2026 Apr 30;17:5903. doi: 10.1038/s41467-026-72399-4 (PMC13338143; doi:10.1038/s41467-026-72399-4)
Supplement: Supplementary file 1 — Supplementary information [file 41467_2026_72399_MOESM1_ESM.pdf]

Supplementary information for

Managed rainforests support higher carbon density and sequestration in the Congo Basin

Le Bienfaiteur Sagang*, Ricardo Dalagnol, Lee White, Stephanie George-Chacon, Samuel Favrichon, Shuang Li, Fabien Wagner, Zhihua Liu, Dafeng Zhang, Alfred Ngomanda, Vincent Medjibe, Bonaventure Sonké, Nicolas Barbier, Elsa M. Ordway, Sassan Saatchi
*Correspondence to: sagang.bienfaiteur@yahoo.fr

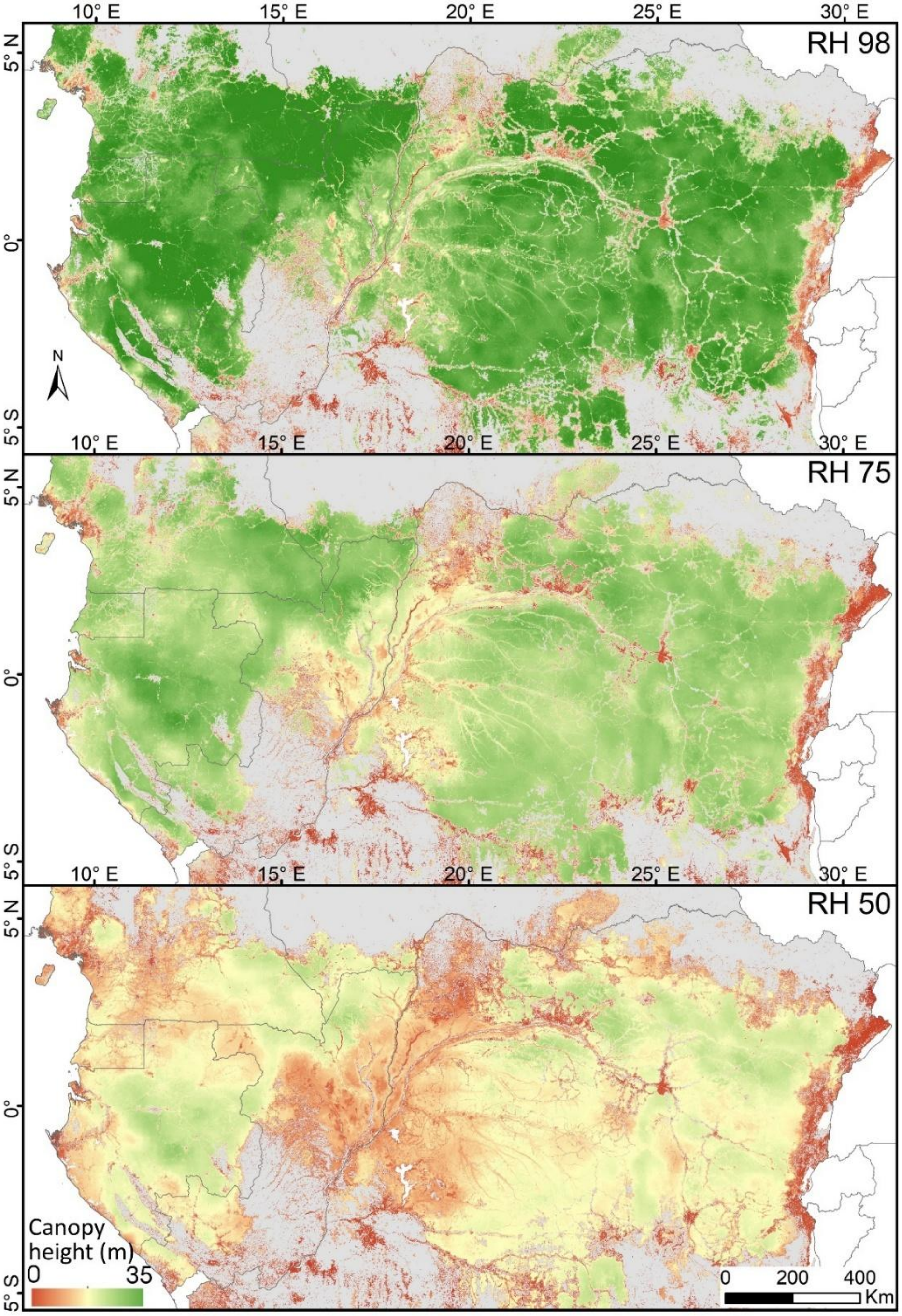

Figure S1: Wall-to-wall GEDI relative canopy height (m) predictions over the Congo Basin rainforest for the year circa 2020 at 100 m spatial resolution. RH98 = top; RH75 = middle, RH50 = bottom.

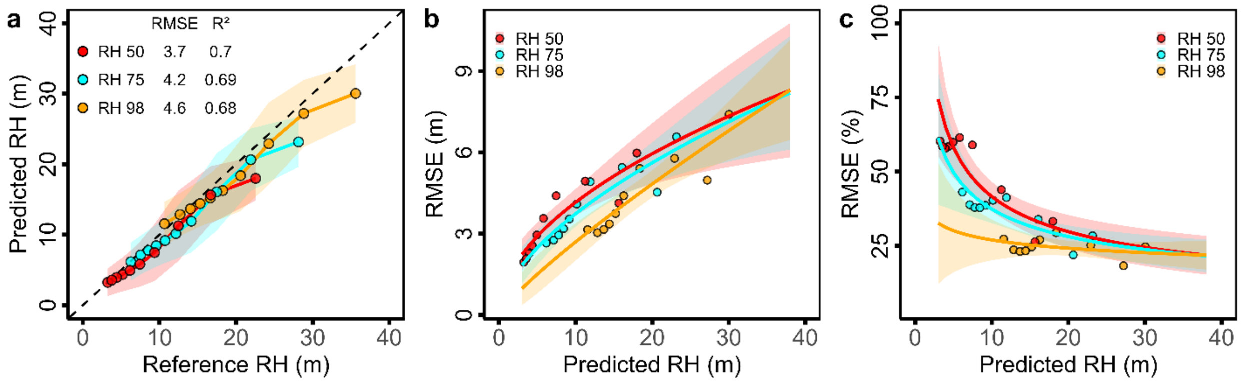

Figure S2: Validation of GEDI relative canopy height (RH50; RH75 and RH98) predictions over the Congo Basin. (a) Scatter plot depicting the relation between RH predictions and 20% validation subset of GEDI samples, averaged across successive 10% percentile bins, with 95% confidence intervals represented by colored polygons. (b and c) Root mean squared error (RMSE) of each RH prediction, expressed in absolute (m) or relative (%) terms, with the function used to propagate model uncertainty.

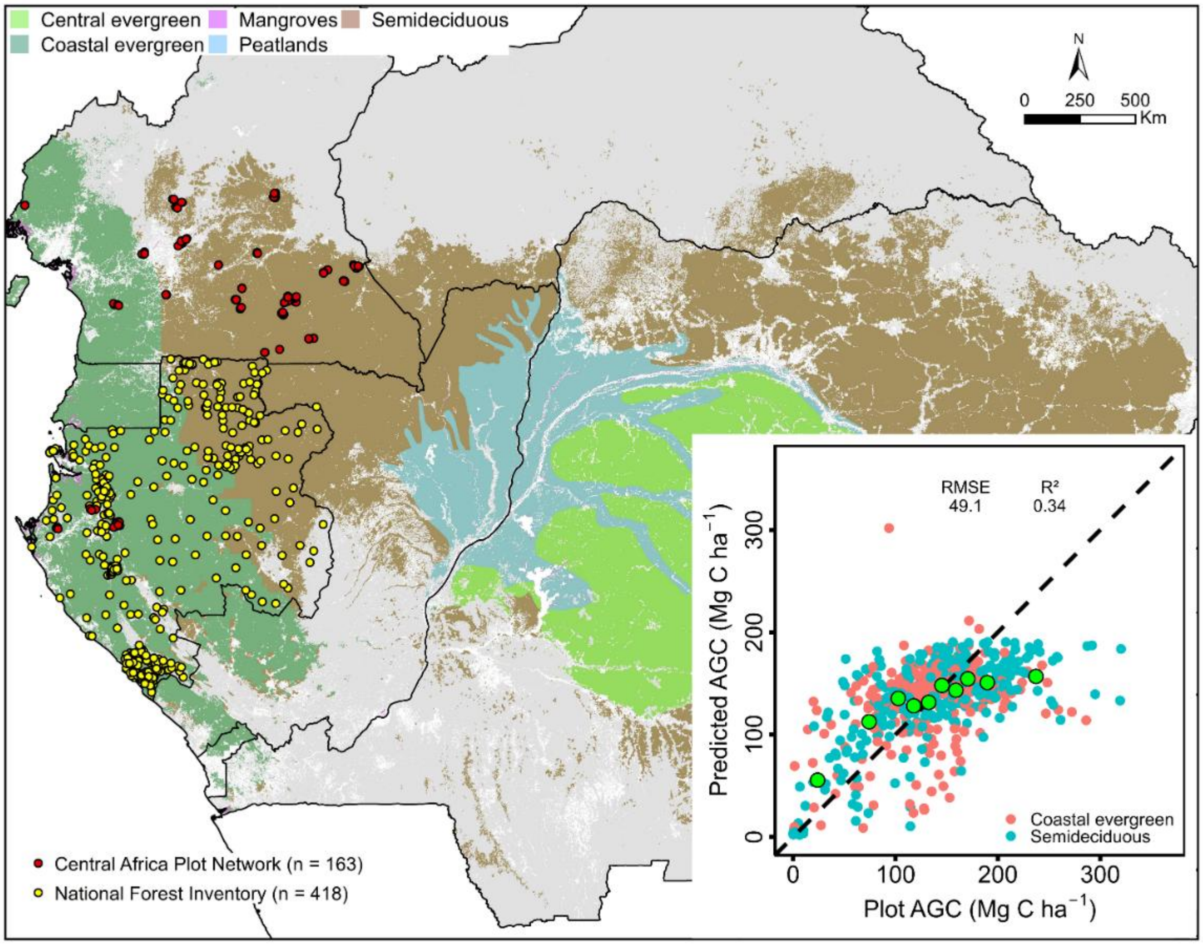

Figure S3: Validation of your AGC map with 418 plots from the National Field Inventory (NFI) data from Gabon and 163 plots from the Central African plot network.

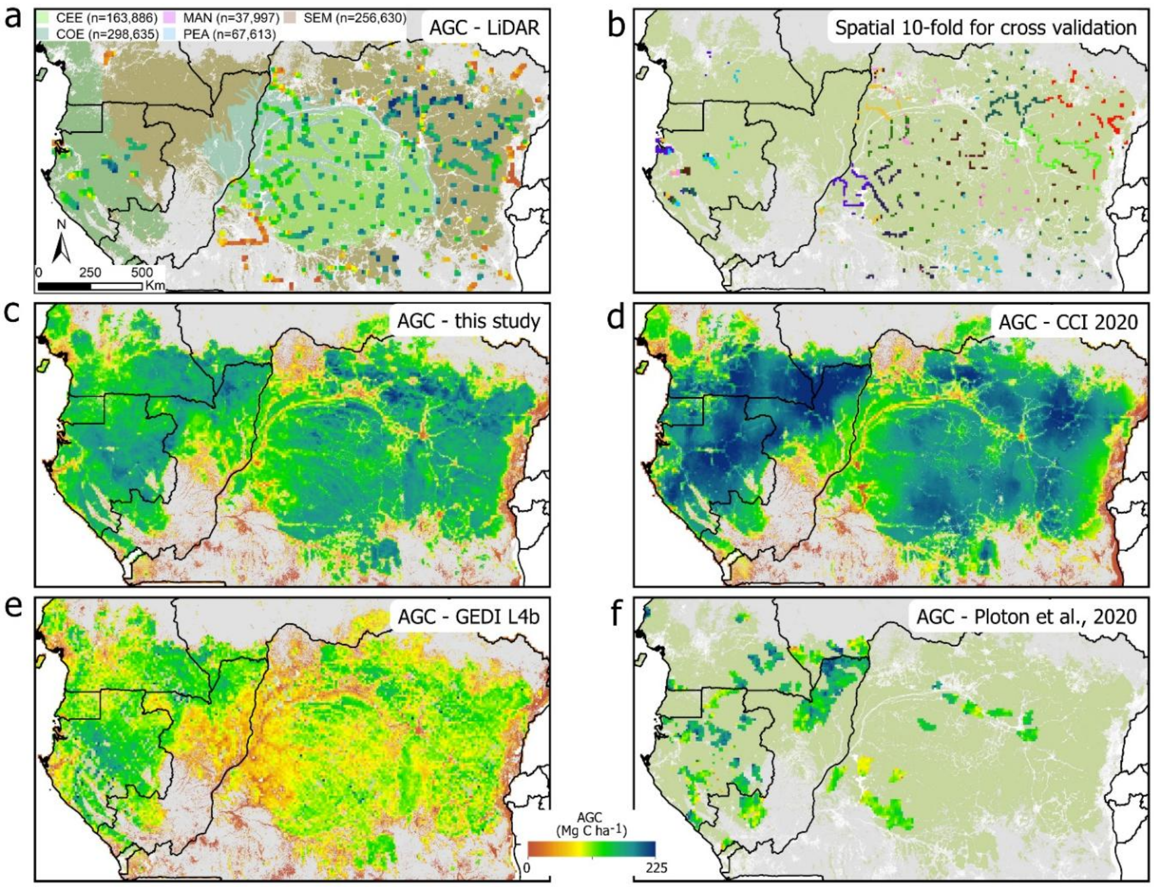

Figure S4: Comparison in the spatial distribution of above ground carbon (Mg C ha^{-1}) from different products over the Congo Basin rain forest all aggregated at 10 km resolution for visual illustration. (a) LiDAR-derived AGC samples over the different forest types in background and the number of 1-ha samples for each forest type (~ 825,000). (b) Spatial pattern of the 10 folds used for spatial cross validation. (c) This study. (d) ESA CCI 2020. (e) GEDI L4d. (f) management inventories in the Congo Basin (CoFor-AGB[101]).

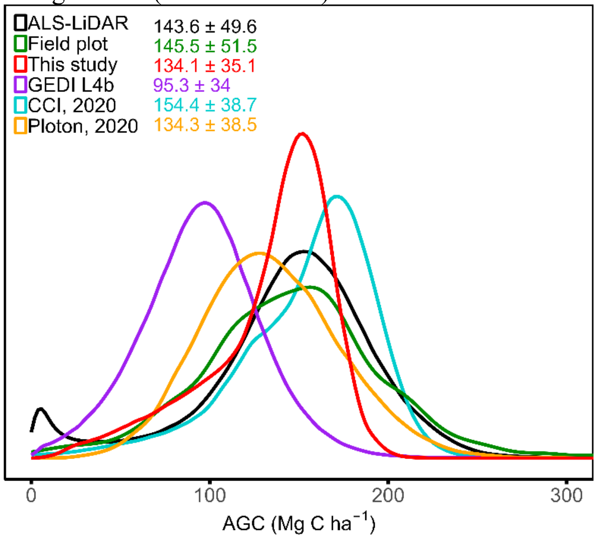

Figure S5: Distributions of the aboveground carbon density (AGC, Mg C ha^{-1}) with the respective means from the different maps and additional 673 1-ha plots data including 92 plots from Xu et al.[47], over DRC, 163 plots from the Central African plot network (http://www.cafriplot.net) and 418 plots from the National Field Inventory (NFI) data from Gabon.

Table S1: Aboveground carbon accumulation rates (Mg C ha^{-1}y^{-1}) within recovering forests from clearing and degradation across land-uses and chronosequence, with 10 years intervals following the last disturbance.

Age (years)	Concessions				Protected				Unprotected			
	CEE	COE	SEMI	PEA	CEE	COE	SEMI	PEA	CEE	COE	SEMI	PEA
	Forest clearing											
≤10	2.71 ±0.14	2.98 ±0.23	3.26 ±0.19	2.24 ±0.18	3.20 ±0.16	2.22 ±0.20	3.50 ±0.19	1.13 ±0.13	2.87 ±0.15	3.28 ±0.15	3.12 ±0.15	2.32 ±0.15
10-20	1.44 ±0.11	1.39 ±0.18	1.63 ±0.13	1.44 ±0.12	1.46 ±0.11	1.34 ±0.25	1.77 ±0.12	0.88 ±0.12	1.27 ±0.11	1.52 ±0.12	1.52 ±0.10	1.27 ±0.12
20-30	1.1 ±0.15	1.01 ±0.25	1.23 ±0.16	1.18 ±0.14	1.11 ±0.14	1.07 ±0.42	1.32 ±0.13	0.79 ±0.11	0.71 ±0.19	0.91 ±0.13	1.13 ±0.11	0.98 ±0.14
>30	0.82 ±0.28	0.72 ±0.41	0.89 ±0.29	0.92 ±0.31	0.74 ±0.24	0.84 ±0.73	0.94 ±0.24	0.69 ±0.3	0.71 ±0.19	0.63 ±0.22	0.81 ±0.2	0.74 ±0.26
	Forest degradation											
≤10	2.57 ±0.26	2.66 ±0.16	2.32 ±0.23	2.61 ±0.26	2.31 ±0.25	2.98 ±0.27	2.33 ±0.21	2.33 ±0.28	2.14 ±0.23	3.04 ±0.14	2.41 ±0.22	2.33 ±0.23
10-20	0.97 ±0.17	0.86 ±0.12	0.74 ±0.16	0.97 ±0.19	0.76 ±0.17	1.21 ±0.19	0.75 ±0.14	0.85 ±0.21	0.71 ±0.15	1.09 ±0.10	0.82 ±0.14	0.85 ±0.15
20-30	0.65 ±0.22	0.55 ±0.15	0.47 ±0.19	0.65 ±0.26	0.49 ±0.21	0.84 ±0.25	0.48 ±0.17	0.57 ±0.26	0.46 ±0.18	0.72 ±0.12	0.53 ±0.17	0.56 ±0.19
>30	0.42 ±0.33	0.34 ±0.2	0.29 ±0.26	0.42 ±0.38	0.30 ±0.3	0.56 ±0.38	0.29 ±0.23	0.36 ±0.38	0.28 ±0.25	0.46 ±0.18	0.33 ±0.24	0.36 ±0.28

Table S2: Aboveground carbon accumulation rates (ΔAGC, Mg C ha^{-1}y^{-1}) and density (AGC, Mg C ha^{-1}) for recovering forests across land-uses and chronosequence, with 10-year intervals following the last disturbance.

Age (years)	Certified concessions		Uncertified concessions		Protected		Unprotected	
	ΔAGC	AGC	ΔAGC	AGC	ΔAGC	AGC	ΔAGC	AGC
	Forest clearing							
≤10	2.95 ±0.2	32.56 ±11.9	2.64 ±0.17	25.99 ±10.5	2.51 ±0.17	24.76 ±13.9	2.9 ±0.16	34.76 ±10.8
10-20	1.49 ±0.11	53.2 ±10.4	1.46 ±0.14	45.37 ±8.8	1.36 ±0.15	42.43 ±16.3	1.39 ±0.11	54.90 ±8.5
20-30	1.12 ±0.17	66.34 ± 8.8	1.14 ±0.18	58.49±8.9	1.06±0.2	54.34±9.9	1.03 ±0.13	67.33±7.08
>30	0.82 ±0.33	84.78 ± 16.6	0.85 ± 0.33	77.53±16.4	0.81 ±0.38	71.81±18.9	0.74±0.23	84.44±12.1
	Forest degradation							
≤10	2.5 ±0.24	77.14 ±19.7	2.58 ±0.22	66.95 ±19.8	2.49 ±0.26	60.78 ±12.7	2.48 ±0.21	63.1 ±9.1
10-20	0.84 ±0.16	91.43 ±17.7	0.92 ±0.16	82.21 ±17.5	0.89 ±0.18	75.33 ±8.4	0.87 ±0.13	77.84 ±7.1
20-30	0.55 ±0.21	98.42±10.6	0.61 ±0.21	89.92±10.4	0.59 ±0.23	82.72±11.4	0.57 ±0.17	85.21±8.6
>30	0.34±0.29	106.62±14.8	0.39±0.29	99.35±15.1	0.38±0.33	91.77±16.4	0.36±0.24	94.09±12.3

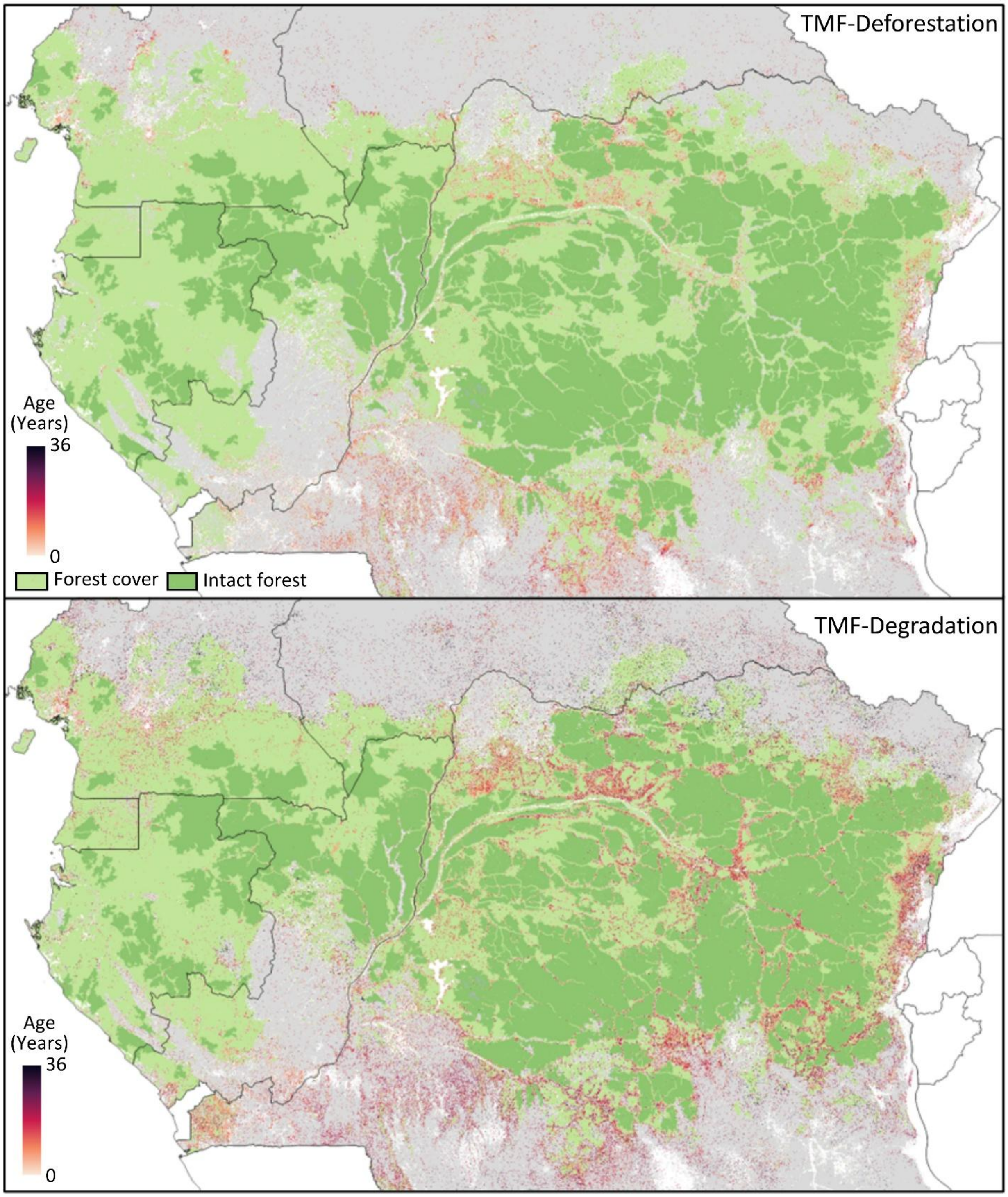

Figure S6: Age in 2020 of recovering forests from clearing (top) and degradation (bottom) as defined in the Tropical Moist Forest cover change dataset[21] with old growth forests in 2020 displayed as dark green.

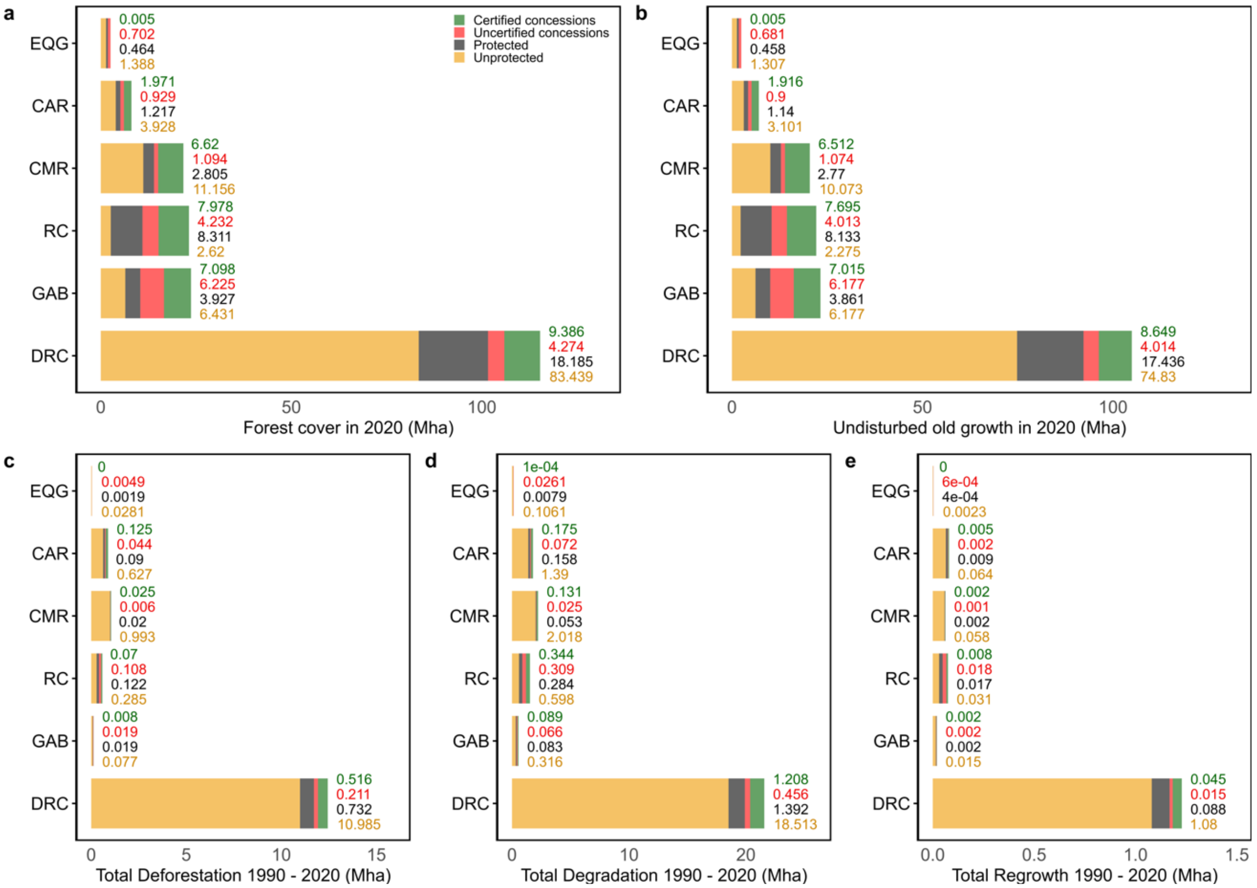

Figure S7: Comparison of total TMF cover change (1990-2020) across land-use for the Congo Basin countries.

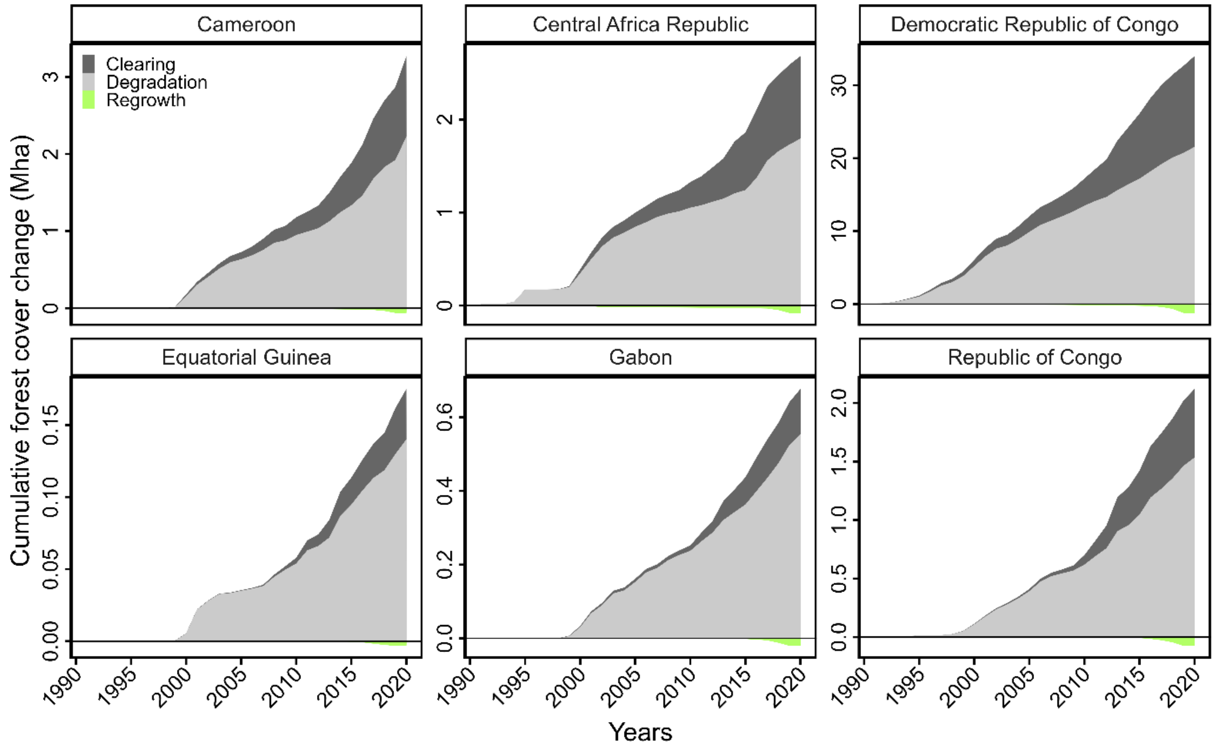

Figure S8: Comparison of annual TMF cover change (1990-2020) for the Congo Basin countries.

Table S3: Forest cover in 2020 (Mha), aboveground carbon density (Mg C ha^{-1}), stocks (Tg C) and changes (Tg C yr^{-1}) across land-use for the Congo Basin countries from 1990 to 2020. The net AGC changes, emissions and sinks are reported as the long-term average fluxes.

Country	land-use	Forest cover	AGC	AGC stock	Annual rate between [1990-2020]		
					AGC Emissions	AGC Removals	Net AGC change
Cameroon	Certif. concessions	6.7	148.1 ±18.1	992.3	0.3	-4	-3.7
	Uncertif. concessions	1.1	145.9 ±18.3	160.5	0.1	-0.6	-0.5
	Protected	2.8	134.3 ±36.4	376.0	0.2	-1.9	-1.7
	Unprotected	11.1	118.6 ±30.6	1316.5	6.2	-6.3	-0.1
Democratic Republic of Congo	Certif. concessions	9.4	144.6 ±29.3	1359.2	4.3	-6.5	-2.2
	Uncertif. concessions	4.3	137 ±30.2	589.1	1.5	-3.1	-1.6
	Protected	18.2	136.6 ±35.3	2486.1	4.6	-11.4	-6.8
	Unprotected	83.4	132.9 ±38.6	11083.9	49.7	-46.8	2.9
Equatorial Guinea	Certif. concessions	0.005	136.8 ±8.4	0.7	0.003	-0.001	0.002
	Uncertif. concessions	0.7	130.8 ±20.6	91.6	0.01	-0.3	-0.3
	Protected	0.5	126.6 ±29.6	63.3	0.01	-0.3	-0.3
	Unprotected	1.4	129.7 ±20.4	181.6	0.3	-0.9	-0.6
Gabon	Certif. concessions	7.1	151.5 ±16.6	1075.7	0.2	-4.1	-3.9
	Uncertif. concessions	6.2	148.4 ±17.9	920.1	0.2	-2.8	-2.6
	Protected	4	137.9 ±32.4	551.6	0.3	-2.7	-2.4
	Unprotected	6.4	137.6 ±29.9	880.6	0.7	-3.4	-2.7
Central Africa Republic	Certif. concessions	2	144.6 ±33.1	289.2	0.8	-1.3	-0.5
	Uncertif. concessions	1	147.6 ±28.1	147.6	0.3	-0.6	-0.3
	Protected	1.2	125.1 ±28.9	150.1	0.5	-0.9	-0.4
	Unprotected	3.9	84.1 ±24.11	328.0	2.5	-2.3	0.2
Republic of Congo	Certif. concessions	8	144.4 ±24.3	1155.2	0.9	-4.8	-3.9
	Uncertif. concessions	4.2	133.3 ±28.9	559.9	0.8	-2.6	-1.8
	Protected	8.3	125.2 ±30.6	1039.2	0.9	-5	-4.1
	Unprotected	2.6	113.1 ±34.8	294.1	1.1	-1.4	-0.3
