# Supplementary material for: Managed rainforests support higher carbon density and sequestration in the Congo Basin
Source: Nat Commun. 2026 Apr 30;17:5903. doi: 10.1038/s41467-026-72399-4 (PMC13338143; doi:10.1038/s41467-026-72399-4)
Supplement: Supplementary file 3 — Transparent Peer Review file [file 41467_2026_72399_MOESM3_ESM.pdf]

nature portfolio

Peer Review File

Managed rainforests support higher carbon density and sequestration in the Congo Basin

Corresponding Author: Dr Le Bienfaiteur Sagang

This file contains all reviewer reports in order by version, followed by all author rebuttals in order by version.

Version 0:

Reviewer comments:

Reviewer #1

(Remarks to the Author)
This manuscript presents a high-resolution, data-driven quantification of above-ground carbon (AGC) density and its variation across land-use classes in the Congo Basin rainforest. The study integrates airborne LiDAR calibration, GEDI canopy metrics, and multi-source satellite predictors with 30 years of forest cover dynamics to produce one of the most detailed regional AGC assessments to date. The work is methodologically rigorous, regionally novel, and policy-relevant. It represents a valuable contribution to tropical carbon accounting and land-use policy research. However, the current version would benefit from revisions to strengthen scientific depth and clarify causal mechanisms.

(Remarks on code availability)
This manuscript presents a high-resolution, data-driven quantification of above-ground carbon (AGC) density and its variation across land-use classes in the Congo Basin rainforest. The study integrates airborne LiDAR calibration, GEDI canopy metrics, and multi-source satellite predictors with 30 years of forest cover dynamics to produce one of the most detailed regional AGC assessments to date. The work is methodologically rigorous, regionally novel, and policy-relevant. It represents a valuable contribution to tropical carbon accounting and land-use policy research. However, the current version would benefit from revisions to strengthen scientific depth and clarify causal mechanisms.
Abstract
(1) Line 20: Change “by developing AGC density map” into “by developing an AGC density map” for grammatical correctness.
(2) Line 27: “dominated my 98% removals” should be “dominated by 98% of removals.”
1. Main
(1) Line 33: “The Congo basin rainforest, the second-largest expanse of tropical forest globally is home…” should be that “… globally, is home…”
(2) Line 45: “Long-term land-use management that…are essential to…” should be that “… is essential…”
(3) Line 61: “The airborne and spaceborne Light Detection and Ranging (LiDAR) technologies offers…” should be that “...technologies offer…”
(4) Line 71: “the democratization of nearly four decades of optical satellite image archives from Landsat (USGS, 2020), allows for…” should be that “the democratization of nearly four decades of optical satellite image archives from Landsat (USGS, 2020) allows for…”
(5) Line 82: “including …, protected unprotected areas” should be that “and protected unprotected areas”
2. Results
(1) Line 96: The description of model performance (R2= 0.59; RMSE = 27.94 Mg C ha-1 and bias = -4.46) could be misleading. It appears that statistic values represent the mean of the model performance across the five main forest-type strata rather than the accuracy over the entire Congo Basin in Figure 1a. This distinction is important because it affects how model robustness and generalizability are interpreted. I suggest that you should make it clear
(2) Line 117: The sentence “An ANOVA test shows that central evergreen forests had the highest AGC density…” reports group means but doesn’t specify whether differences were statistically significant (e.g., $p < 0.05$) or which groups differ according to post hoc tests.
(3) Line 125: We found a total of 26.14 Pg C, with managed (logging + protected) forests = 12.04 Pg C ≈ 46%, and unprotected forests = 14.1 Pg C ≈ 54%. However, the origin text only focuses on managed forests, giving the misleading impression that they dominate. Figure 1c shows that unprotected forests have the highest total live AGC storage, this should be clearly stated in the text.

(4) Lines 128-135: This paragraph deserves further polishing to improve readability. For example, “However, an exception was observed…”, “logged forests had 26.64% more AGC density” can express that “logged forests exhibited 26.64% higher AGC density…”. Additionally, ensure absolute values are followed by units (e.g. Mg C ha-1).
(5) Line 154: Please confirm that “Figure 3a”? I think it may be Figure 3b.
(6) Line 155: The text states that the AGC accumulation rate in older recovering forests following forest clearing is “2.6 times higher” than in degraded areas. However, based on the values provided (0.81 ± 0.32 vs. 0.37 ± 0.28 Mg C ha^{-1} yr^{-1}), the ratio is approximately 2.2 (i.e., 0.81 / 0.37 ≈ 2.19). Please verify the calculation of the “2.6 times”. For clarity and precision, it is recommended to report the exact ratio derived from the data or to rephrase the sentence to avoid any ambiguity.
(7) Line 169: “… and secondary forests (-16.91 ± 2.38 Tg C; 0.65%)”. The Figure 4 doesn't show the change of AGC in secondary forests. Is this “regrowth”? I recommend the expression of the text is consistent with this statement of plot.
3. Discussion
(1) Lines 226-232: While the discussion lists multiple reasons why managed forests show higher AGC density, the presentation reads as a compilation rather than a cohesive argument. The authors are encouraged to reorganize these explanations into a more causal narrative, and to provide more quantitative or referenced evidence supporting each mechanism. This will improve the persuasive strength of the interpretation.
(2) The finding that forest management contributes approximately 98.77% of the net carbon sink in the Congo Basin is highly significant, yet it appears buried in the middle of the section (e.g. line 270). This result should be more prominently featured and explicitly linked to implications for climate policy and forest governance. The discussion should draw a stronger connection between this quantitative outcome and the broader argument about the role of management in carbon mitigation.
(3) The concluding paragraphs combine several topics—biodiversity, REDD+, economic incentives, and governance—making the ending somewhat diffuse. I encourage that the final paragraph can split into two paragraphs.

Reviewer #2

(Remarks to the Author)
Managed forests support higher carbon density and sequestration in the Congo basin

This study developed an aboveground live biomass (AGC) map in the Congo basin in 2020 and integrated it with forest cover loss data from 1990 to 2020 to estimate carbon emissions and removals using space-for-time approaches. The topic of this study is interesting. However, I have three major concerns about this study.

1. The methodology for estimating the AGC map in the Congo basin is unclear. Specific and localized machine learning (ML) models were developed for the AGC estimation in each forest type across the Congo basin. The comparison of AGC emissions and removals among these forest types could have high uncertainties. The estimates of AGC emissions and removals from forest disturbances had many assumptions due to limitations of the space-for-time approaches and the spatial variability of forests in the Congo basin, and it is hard to assess the reasonability of these assumptions.

2. The Discussion section is weak and lacks strong data analysis or evidence to support the major findings of this study.

3. The writing of this paper has obvious errors and needs to be carefully double-checked. For example,
1) Line 27, “dominated my 98% removals from managed and intact forests”
2) Line 82 “protected unprotected areas”
3) Line 95-104 in the Results and Line 205-210 in the Discussion showed repeated writing but different or opposite conclusions.
4) The numbering of figures and tables in the manuscript was messed up.

Minor comments:
1. What is the definition of managed forest in this study?

2. The method and process for calculating wall-to-wall canopy height are unclear.

3. The maps of the ages of recovering forests and canopy height are critical data in this study. Please add the spatial maps of these two datasets.

(Remarks on code availability)
I could not open the code link.

Reviewer #3

(Remarks to the Author)
The manuscript is very well written. The topic is relevant because it supports the discussion on pros and cons of forest management with high-quality information. The applied methods are well explained and the discussion section is well structured.

I recommend the manuscript for publication.

minor edits:
l. 27: I assume 'my' should be 'by'.
l. 514: I assume the link will be provided after publication.

References: some of the cited papers do not show a publisher or a webpage. (Example Kauffman Donato: Kauffmann, J. B. & Donato, D. Protocols for the measurement, monitoring and reporting of structure, biomass and carbon stocks in mangrove forests. Working Paper 86. (CIFOR (2012)).)

(Remarks on code availability)
the code is not yet available.

Version 1:

Reviewer comments:

Reviewer #1

(Remarks to the Author)
I have carefully reviewed the revised version of the manuscript and the authors' detailed responses to the previous comments. Overall, I am very satisfied with the revisions and commend the authors for their thorough and thoughtful efforts in addressing the reviewer concerns. The authors have responded comprehensively and constructively to all major and minor comments, and the manuscript is now scientifically robust, clearly presented, and suitable for publication. I have no further substantive comments and fully support its acceptance.
That said, before final publication, the manuscript would benefit from one final round of careful language polishing, particularly to ensure consistent use of verb tense throughout the Abstract and main text. At present, the Abstract occasionally mixes present and past tense within the same logical sequence (e.g., "drives," "remain," "underscore" versus "exhibited," "remained," "dominated"). Addressing these minor grammatical and stylistic issues will further improve clarity and readability.

(Remarks on code availability)
I was able to access the code repository via the provided link. The code is written in R and uses .fst files for data storage and processing, which is suitable for efficient handling of large datasets. The scripts appear to follow a logical structure for reading and processing the data.

However, the repository does not currently include a README file or equivalent documentation describing package dependencies, data structure, or instructions for running the code. As a result, while the code itself is accessible and readable for users familiar with R, its reproducibility and reusability by the broader community would be improved by the inclusion of basic documentation. I recommend that the authors add a short README outlining the required packages, input data formats, and execution workflow prior to publication.

Reviewer #2

(Remarks to the Author)
The authors have addressed my concern. I am happy to accept this paper for publication.

(Remarks on code availability)

Open Access This Peer Review File is licensed under a Creative Commons Attribution 4.0 International License, which permits use, sharing, adaptation, distribution and reproduction in any medium or format, as long as you give appropriate credit to the original author(s) and the source, provide a link to the Creative Commons license, and indicate if changes were made.
In cases where reviewers are anonymous, credit should be given to 'Anonymous Referee' and the source.
The images or other third party material in this Peer Review File are included in the article's Creative Commons license, unless indicated otherwise in a credit line to the material. If material is not included in the article's Creative Commons license and your intended use is not permitted by statutory regulation or exceeds the permitted use, you will need to obtain permission directly from the copyright holder.
To view a copy of this license, visit https://creativecommons.org/licenses/by/4.0/

RESPONSE TO REFEREES

We thank the reviewers for the time and efforts devoted to improving our manuscript through constructive comments and suggestions. In response, we substantially revised the manuscript, adding more details on the methodological workflow underlying each result and uncertainties, thereby strengthening the robustness of our findings. We also improved the discussion by removing redundant content and reinforcing the narrative with stronger causal arguments and additional quantitative evidence from both our analysis and the literature. We believe the manuscript now meets the highest standards of scientific rigor for publication in Nature Communications. We provide below our point-by-point responses.

Reviewer #1 (Remarks to the Author):

This manuscript presents a high-resolution, data-driven quantification of above-ground carbon (AGC) density and its variation across land-use classes in the Congo Basin rainforest. The study integrates airborne LiDAR calibration, GEDI canopy metrics, and multi-source satellite predictors with 30 years of forest cover dynamics to produce one of the most detailed regional AGC assessments to date. The work is methodologically rigorous, regionally novel, and policy-relevant. It represents a valuable contribution to tropical carbon accounting and land-use policy research. However, the current version would benefit from revisions to strengthen scientific depth and clarify causal mechanisms.

Reviewer #1 (Remarks on code availability):
Abstract
(1) Line 20: Change "by developing AGC density map" into "by developing an AGC density map" for grammatical correctness.
Updated. Now the sentence reads "In this study, we evaluate the variation of aboveground live carbon (AGC) stocks and fluxes across land-use gradients in its rainforests by developing an AGC density map for the year 2020 and integrating it with high-resolution forest cover change data spanning 30 years (1990-2020) to quantify carbon emissions and removals." Line 20-23

(2) Line 27: "dominated my 98% removals" should be "dominated by 98% of removals."
Updated. Now the sentence reads "Old growth forests dominated the total AGC removals across land-uses (84%) with the Congo Basin rainforest functioning as a net AGC sink at -37.5 ±4.8 TgCyr-1, primarily driven by managed land-uses (99%) including logging concessions (-21.3 ±2.4 TgCyr-1) and protected areas (-15.7 ±2.2 TgCyr-1), while unmanaged areas remained nearly neutral." Line 27-31

1. Main
(1) Line 33: "The Congo basin rainforest, the second-largest expanse of tropical forest globally is home…" should be that "…globally, is home…"
Updated. Now the sentence reads "The Congo Basin rainforest, the second-largest expanse of tropical forest globally, is home to an estimated 7,000 endemic plant species (Sosef et al., 2017) as well as iconic animal biodiversity (WWF, 2024) including bonobos, chimpanzees, gorillas, forest elephants, and okapi, and sustains the livelihoods of millions of people (FAO, 2010)." Line 35-38

(2) Line 45: "Long-term land-use management that…are essential to…" should be that "… is essential…"

Updated. Now the sentence reads “Long-term land-use management that safeguards forest resources and biodiversity while promoting sustainable income revenues is essential to successfully integrate the Congo Basin rainforest into global change adaptation and mitigation efforts (Bele et al., 2015).” Line 47-49

(3) Line 61: “The airborne and spaceborne Light Detection and Ranging (LiDAR) technologies offers…” should be that “...technologies offer…”
Updated. Now the sentence reads “Airborne and spaceborne Light Detection and Ranging (LiDAR) technologies offer an opportunity to sample across functional and structural gradients at climate mitigation policy-relevant scales (Bossy et al., 2025; Dubayah et al., 2021; Ganivet and Bloomberg, 2019; Kumar et al., 2015), and interpolate forest measurements using wall-to-wall satellite products and machine learning (ML) techniques (Lang et al., 2019; Potapov et al., 2021; Sagang et al., 2020).” Line 63-67

(4) Line 71: “the democratization of nearly four decades of optical satellite image archives from Landsat (USGS, 2020), allows for…” should be that “the democratization of nearly four decades of optical satellite image archives from Landsat (USGS, 2020) allows for…”
Updated. Now the sentence reads “In addition, the democratization of nearly four decades of optical satellite image archives from Landsat (USGS, 2020) allows for consistent, long-term characterization of changes in tropical moist forest (TMF) cover and the tracking of TMF gains and losses (Hansen et al., 2013; Vancutsem et al., 2021) at moderate-spatial resolution (30 m), making it ideal for historical trend analysis (Heinrich et al., 2023, 2021).” Line 73-78

(5) Line 82: “including …, protected unprotected areas” should be that “and protected unprotected areas”
Updated. Now the sentence reads “We analyzed AGC distribution across the main forest types (central and coastal evergreen, semi-deciduous, peatlands and mangroves; Dinerstein et al., 2017; Réjou-Méchain et al., 2021), and land-use management regimes, including certified and uncertified logging concessions, protected and unprotected areas.” Line 83-86

2. Results
(1) Line 96: The description of model performance (R2= 0.59; RMSE = 27.94 Mg C ha-1 and bias = -4.46) could be misleading. It appears that statistic values represent the mean of the model performance across the five main forest-type strata rather than the accuracy over the entire Congo Basin in Figure 1a. This distinction is important because it affects how model robustness and generalizability are interpreted. I suggest that you should make it clear
Thank you for raising this point. We have updated the sentence and we believe the distinction is clear now “The AGC estimates derived from our Gradient Boosted Regression Trees (GBRT) models, trained on ~ 825,000 ha of reference LiDAR-derived AGC data outperformed existing global and regional products across forest types (Figure 1, see the Materials and Methods), with averaged $R^2 = 0.6$; RMSE = 28 Mg C ha-1 and bias = -4.5 (Figure 1a).” Line 100-103

(2) Line 117: The sentence “An ANOVA test shows that central evergreen forests had the highest AGC density…” reports group means but doesn’t specify whether differences were statistically significant (e.g., $p < 0.05$) or which groups differ according to post hoc tests.
We have provided more information on the significance of the result with the p-value “An ANOVA test revealed a significant difference in mean AGC across forest types (p-value <2e-16), with a Tukey post hoc test showing that central evergreen forests had the highest AGC density (146.7 ± 22.2 Mg C ha-1, Figure 1c) followed by coastal evergreen (137.5 ± 26.3 Mg C ha-1) and

semideciduous forests (131.1 ± 39.4 Mg C ha-1), with lowest values found in mangroves (50.3 ± 34 Mg C ha-1).” Line 125-129

(3) Line 125: We found a total of 26.14 Pg C, with managed (logging + protected) forests = 12.04 Pg C ≈ 46%, and unprotected forests = 14.1 Pg C ≈ 54%. However, the origin text only focuses on managed forests, giving the misleading impression that they dominate. Figure 1c shows that unprotected forests have the highest total live AGC storage, this should be clearly stated in the text.
The sentence has been updated accordingly “We estimated at 26.1 ± 3.2 Pg C the total live AGC stock in the Congo Basin rainforests in 2020, with 54.1% (14.1 ± 0.6 Pg C) found within unprotected forests and 46.1% (12.1 ± 0.9 Pg C) shared between logging concessions (7.3 ± 0.56 Pg C) and protected areas (4.7 ± 0.6 Pg C).” Line 133-136

(4) Lines 128-135: This paragraph deserves further polishing to improve readability. For example, “However, an exception was observed…”, “logged forests had 26.64% more AGC density” can express that “logged forests exhibited 26.64% higher AGC density…”. Additionally, ensure absolute values are followed by units (e.g. Mg C ha-1).
The paragraph has been updated accordingly “On average, AGC in selectively logged forests was 7.5% lower than in old growth forests (Figure 2), with differences ranging from 4.9% in coastal evergreen forests (Figure 2b) to 9.5% in semideciduous forests (Figure 2c). Peatlands were an exception, where logged forests exhibited 26.6% higher AGC density (158.3 ± 26.3 Mg C ha-1) than old growth forests (125 ± 34.7 Mg C ha-1). Forests affected by unmanaged disturbances including slash-and-burn showed the lowest AGC density, reaching 50.1% less AGC than old growth forests.” Line 138-143

(5) Line 154: Please confirm that “Figure 3a”? I think it may be Figure 3b.
Updated to Figure 3b. Line 160

(6) Line 155: The text states that the AGC accumulation rate in older recovering forests following forest clearing is “2.6 times higher” than in degraded areas. However, based on the values provided (0.81 ± 0.32 vs. 0.37 ± 0.28 Mg C ha^{-1} yr^{-1}), the ratio is approximately 2.2 (i.e., 0.81 / 0.37 ≈ 2.19). Please verify the calculation of the “2.6 times”. For clarity and precision, it is recommended to report the exact ratio derived from the data or to rephrase the sentence to avoid any ambiguity.
We have updated the rate of carbon recovery and adjusted the sentence accordingly “Older (>30 years) recovering forests displayed an average annual AGC accumulation of 0.6 ± 0.4 Mg C ha-1 yr-1, with the rate of recovery being 2.2 times higher following forest clearing (0.8 ± 0.3 Mg C ha-1 yr-1) compared to forest degradation (0.4 ± 0.3 Mg C ha-1 yr-1). Line 163-165

(7) Line 169: “… and secondary forests (-16.91 ± 2.38 Tg C; 0.65%)”. The Figure 4 doesn't show the change of AGC in secondary forests. Is this “regrowth”? I recommend the expression of the text is consistent with this statement of plot.
The term “secondary forest” refers to regrowth from clearing. We have updated this expression throughout the text to be consistent with the figure. The sentence now reads like this “Old growth forests dominated the AGC removals across all land use (-2,861.9 ± 314.8 Tg C, 83.8%) followed by degraded (-536.8 ± 64.6 Tg C; 15.7%) and forests recovering from clearing (-16.9 ± 2.4 Tg C; 0.7%).” Line 180-182

We have provided Table 1 with the estimates of AGC emissions, and removals from old growth, degraded and recovering forests from clearing (Line 176).

Table 1: AGC removals and emissions over the Congo Basin rainforest between 1990-2020. Removals (negative values, Tg C) include AGC accumulation within old growth, degraded and recovering forests from clearing, while emissions (positive values) include AGC losses from clearing and degradation of degraded and old growth forests. Total and annual net changes (loss – gain, Tg C $year^{-1}$) are aggregates within each land-use over 30 years.

Land-use	AGC Removals			AGC Emissions		Total net change	Annual net change
	Old growth	Degraded	Regrowth	Clearing	Degradation		
Certified concessions	-589 ±64.8	-31.3 ±3.8	-0.6 ±0.1	75.5 ±5.7	120.6 ±9.8	-424.8 ±78	-14.2 ±2.6
Uncertified concessions	-284.6 ±30.3	-15.5 ±1.3	-0.3 ±0.04	36 ±2.7	50.2 ±4.1	-214.3 ±25.2	-7.1 ±0.84
Protected	-627.7 ±70	-35.2 ±5.7	-1.7 ±0.3	86.8 ±6.5	105.58 ±12.5	-472.2 ±57	-15.7 ±1.9
Unprotected	-1360.6 ±143.6	-454.8 ±54.7	-14.3 ±2	904.7 ±67.8	911.3 ±73.8	-13.8 ±3	-0.5 ±0.1
**Total**	**-2861.9 ±314.8**	**-536.8 ±64.6**	**-16.9 ±2.4**	**1103 ±89.3**	**1187.7 ±94.1**	**-1125.1 ±144**	**-37.5 ±4.8**

3. Discussion

(1) Lines 226-232: While the discussion lists multiple reasons why managed forests show higher AGC density, the presentation reads as a compilation rather than a cohesive argument. The authors are encouraged to reorganize these explanations into a more causal narrative, and to provide more quantitative or referenced evidence supporting each mechanism. This will improve the persuasive strength of the interpretation.

We thank the reviewer for this suggestion. The text has been adjusted to bring more evidence and causality to the mechanisms described. "Managed forests within logging concessions support 10% more AGC density compared to other land-uses, with certified logging concessions remaining notably higher AGC than that observed in protected areas. This aligns with a pattern already documented in previous studies in the region from field and remote sensing-based estimates (Medjibe, 2020; Poulsen et al., 2020; Sagang et al., 2024). The higher AGC density observed within logging concessions likely results from the combined effects of how they are established and operated. Unlike concessions, protected areas are established more recently and often on previously logged forests, or were given lower economic priority, making them more vulnerable to illegal land-use activities that negatively affect their AGC densities (Pyhälä et al., 2016). Logging concessions in Central Africa have a long history, beginning in colonial times and continuing through post-independence, initially dominated by European companies. More recently, Asian and North American companies have also established a presence. These concessions are typically located in areas rich with large, harvestable trees (minimum cutting diameter of 70 cm, ATIBT, 2023), inherently supporting high AGC (Bastin et al., 2015; Nasi et al., 2012; Perez et al., 2005; Atyi, 2022Lewis et al., 2013b). Within these areas, the selective logging practice removes only a small number of commercial stems per hectare (on average 0.7-2 trees ha-1), limiting carbon losses below 9% (Dupuis et al., 2025; Medjibe et al., 2013, 2011; Tritsch et al., 2020; Umunay et al., 2019). Our extensive data across the basin confirms this limited impact with logged forests showing on average 7.5% less AGC density (range: 4.9% - 9.5%) than undisturbed forests. Extended rotation cycles of at least 20 years between consecutive harvests, as implemented in Gabon (Pérez et al., 2005; République Gabonaise, 2001), further provide more time for the forest to recover between harvests, at higher rates of post-harvest carbon sequestration (Gourlet-Fleury et al., 2013b; Medjibe, 2020). Finally, the closure of temporary roads within concessions (Kleinschroth et al., 2019) restricts access to logged areas, reducing subsequent

degradation from shifting agriculture, hunting, and unauthorized timber or fuelwood extraction. An exception is observed in central evergreen forests within the Democratic Republic of Congo (DRC), where the AGC density of uncertified logging concessions lacking a sustainable management plan (137 ± 30.2 Mg C ha-1 yr-1) is much closer to unmanaged forests (132.9 ± 38.6 Mg C ha-1 yr-1). This reflects the forestry laws in DRC, that allow a wide range of human activities within concessions, including the expansion of shifting agriculture (Hugel and Mashini, 2012; Tyukavina et al., 2018), unregulated timber harvesting for community needs (Lescuyer et al., 2014; Schure et al., 2011), human settlements (Engunda et al., 2018), and mining (Stoop et al., 2019). As a result, forest clearing and degradation inside concessions in DRC occur at rates two to three times higher than in other Congo Basin countries (Ernst et al., 2013; Kleinschroth et al., 2019)." Line 228-261

(2) The finding that forest management contributes approximately 98.77% of the net carbon sink in the Congo Basin is highly significant, yet it appears buried in the middle of the section (e.g. line 270). This result should be more prominently featured and explicitly linked to implications for climate policy and forest governance. The discussion should draw a stronger connection between this quantitative outcome and the broader argument about the role of management in carbon mitigation.

Thank you for this helpful suggestion. We brought this finding earlier up in the paragraph and provided more arguments to connect this finding with the role of management in carbon mitigation in the Congo basin. The section now reads "Managed forests are responsible for almost all (98.7%) of the net carbon sink of the Congo Basin rainforest, while unmanaged areas remain almost carbon neutral over the past 30 years. This highlights the need for strategic planning for climate change mitigation through nature-based solutions centered on the Congo Basin rainforest to effectively reduce Greenhouse Gas (GHG) emissions, enhance carbon sequestration and maximize the associated environmental and socioeconomic benefits to the community (Roe et al., 2021; Schumacher, 2023; Seddon, 2022). Where national circumstances permit, it is vital to prioritize the protection of old growth forests from deforestation and degradation (Mackey et al., 2020), as they account for a significant 83.8% to the total annual AGC gross removals estimated at 95.4 ± 7.6 Tg C yr-1. However, the Congo Basin rainforest exists within countries grappling with significant development challenges, heavily reliant on the economic benefits these forests provide (Tchatchou et al., 2015; World Bank, 2022). The region is also expected to experience five-fold population growth by the end of this century, with expanded land-use pressure on the remaining old growth forests dominated by large- and small-scale agricultural development (Luckeneder et al., 2021; Ordway et al., 2017; Shapiro et al., 2023). The Republic of Congo and Gabon offer a potential model for effective forest management; each boasting approximately 23 million hectares of forest cover as of 2020 (Figure S7). Most of this forest area is managed, with 52.76% in concessions in the Republic of Congo and 56.26% in Gabon, along with 35.91% and 16.58% designated as protected areas, respectively. These concessions operate under legally binding management plans approved by the government, which adhere to standards comparable to those governing national forests in the United States and are classified as Type 6 protected areas by the International Union for Conservation of Nature (IUCN). Consequently, the net annual AGC sinks in these countries are over 1.3 times greater than the net sink reported in DRC, which recorded -7.7 Tg C per year. DRC, the largest forested country in the region with 115 million hectares of rainforest cover, has only 27.62% of its forests under management. Between 1990 and 2020, DRC experienced the largest reduction of its old growth forests with a decline of 32%, equivalent to 33.7 Mha, (Figure S7 & S8), primarily driven by forest cover loss in unmanaged areas (29.4 Mha).

Gabon and the Republic of Congo also illustrate that effectively managed forests can promote positive biodiversity outcomes (Zwerts et al., 2024). Notably, logging concessions in Gabon provide habitat for approximately 61,822 forest elephants, representing about 65% of the total national estimate (95,110; Laguardia et al., 2021). This highlights the potential of sustainable forest management to address global challenges while serving as a foundation for national development, including the possibility of unlocking new nature-based financial opportunities through carbon markets, biodiversity credits, and climate funding (Nyika and Dinka, 2022). However, financial mechanisms like Reducing Emissions from Deforestation and Forest Degradation (REDD+) have yet to gain traction in the Congo Basin. Recently, the voluntary carbon market faced challenges amidst criticisms regarding result integrity and transparency, alongside shifts in methodologies (VERRA, 2023). Currently, Gabon is the only country in the region with REDD+ results registered on the UNFCCC Lima Hub (CNC, 2021). Despite having validated CO2 emission reductions of 187 million tons from land-use changes, payments have been made for only 3.4 million tons." Line 278-317

(3) The concluding paragraphs combine several topics—biodiversity, REDD+, economic incentives, and governance—making the ending somewhat diffuse. I encourage that the final paragraph can split into two paragraphs.

Thank you for this constructive suggestion. The conclusion has been reworded and split into two sections: a first paragraph (see above, L278-317) focusing on the benefits of good forest governance for biodiversity and carbon credits (e.g. REDD+), and the second paragraph (see below, L318-334) addressing the opportunities for local economies to develop the secondary and tertiary sectors that rely on managed forests.

"Additional economic opportunities from the forest sector, such as removals from recovering forests, afforestation, improved management, and development of secondary or tertiary products from harvested forests can support overall conservation and sustainability of climate and biodiversity core benefits in the region. For example, Gabon enforced a ban on unprocessed timber exports in 2010. Investment in local timber processing, which increases the economic value and created jobs, triggered a threefold increase in forest-sector employment and a 322% rise in its contribution to national GDP by 2022 (Watramez et al., 2023). Extending this model to other Congo Basin countries might improve the economic incentives to preserve standing forests and reduce the threats from future deforestation and degradation. Furthermore, abandoned logging roads often become hotspots for illegal activities such as poaching that greatly affect endangered species and threaten biodiversity worldwide (IPBES, 2019), therefore, safeguards to prevent this must be integrated into forest management and investment plans and environmental and social impact mitigation packages. Forest management in the Congo Basin has great potential to simultaneously mitigate climate change, safeguard forest resources and biodiversity, while enabling sustainable development through income generation. Yet achieving lasting impact will require increased investment in local timber processing as well as improved forest monitoring and governance." Line 318-334

Reviewer #2 (Remarks to the Author):

Managed forests support higher carbon density and sequestration in the Congo basin

This study developed an aboveground live biomass (AGC) map in the Congo basin in 2020 and integrated it with forest cover loss data from 1990 to 2020 to estimate carbon emissions and removals using space-for-time approaches. The topic of this study is interesting. However, I have three major concerns about this study.

1. The methodology for estimating the AGC map in the Congo basin is unclear. Specific and localized machine learning (ML) models were developed for the AGC estimation in each forest type across the Congo basin. The comparison of AGC emissions and removals among these forest types could have high uncertainties. The estimates of AGC emissions and removals from forest disturbances had many assumptions due to limitations of the space-for-time approaches and the spatial variability of forests in the Congo basin, and it is hard to assess the reasonability of these assumptions.

Thank you for raising this point. The details on the methodology for estimating AGC map in the Congo basin is described in the section "Mapping forest structure – Aboveground live biomass." Line 409-430, see below. Localized machine learning models within 20 km × 20 km grid cells were only used to interpolate relative canopy height (RH98, RH75, RH50) from GEDI into wall-to-wall RH maps (see Response below related to Canopy height for more details). The regular GEDI sampling design provided sufficient training data within each grid (minimum 500 GEDI pixels aggregated at 100 m), supporting this localized modeling approach. To generate the AGC map, we used reference LiDAR-derived AGC samples (~825,000 ha; Table 2) available over the region to train specific models to each forest type as the spatial distribution of the training samples could not allow a localized ML approach. This approach has been developed in previous studies in the region (Sagang et al., 2024).

"Aboveground live biomass

We gathered extensive airborne LiDAR-derived aboveground biomass (ALS-AGB; Figure S4 & S5, Table 2) data available across the main forest types within the CB at 100 m (Armston et al., 2016; Rodda et al., 2024; Sagang et al., 2024; Xu et al., 2017) to train GBRT-AGB models specific to each forest type. We excluded ALS-AGB pixels for which a canopy cover loss was recorded between the time of acquisition and 2020, resulting in ~ 825,000 pixels available for training at 1-ha (see Figure S4a for the size of training sample within each forest type).

Table 2: LiDAR-based reference aboveground carbon maps over the Congo basin used in this study

Dataset	Country	Acquisition Year	Area (ha)	AGC (Mg C ha-1)	Relative RMSE (%)	R^2
Xu et al., 2017	Democratic Republic of Congo	2016	582,000	115.32 ± 62.51	20.3	0.82
Rodda et al., 2024	Cameroon, Gabon	2012-2022	44,750	77.78 ± 58.29	15.2	0.89
Armston et al., 2016	Gabon	2016	320,018	102.14 ± 39.43	25.72	0.86

The GBRT models to predict AGB (GBRT-AGB) included the HLS and radar predictors in addition to the predicted RH maps, and the Digital Elevation Model (DEM) from Copernicus. The performance of each GBRT-AGB model was assessed using a spatial cross validation approach. The dataset was split into 10 spatial folds created by clustering the coordinates of the pixels using the k-means algorithm (Figure S4b). The model was trained 10 times and for each iteration, one cluster was set aside to validate the model while the remaining folders were used for training. Training data over PEA and MAN were split into 5 folds considering their smaller extent to keep a sufficient size of data for validation (≥ 500 pixels). We compared our results with 1) the AGB product from the European Space Agency (ESA) through the Climate Change Initiative (CCI) version 4 (Santoro and Cartus, 2023); 2) the AGB product from GEDI L4B (Dubayah et al., 2022); 3) the aggregated AGB map from forest management inventory data in the Congo Basin (CoFor-AGB, Ploton et al. (2020) and 4) the AGB measurements from 673 1-ha plots from the cafriplot

network and the national forest inventory (NFI) in Gabon. We used a factor of 0.456 to convert AGB to carbon density (AGC) and an ANOVA test was applied to compare the mean AGC density across management and forest types. The total live AGC stock was obtained by multiplying the AGC density with the total forest area in 2020 as determined by TMF forest cover." Line 414-430

Although we acknowledge the potential limitations of the space-for-time approach, we note that this method has proven effective for estimating AGC accumulation rates across forest types using remote sensing data in tropical forests (Heinrich et al., 2021, 2023). We considered comparing AGC accumulation across forest types considering their influence on carbon fluxes (Muller-Landau et al., 2021). AGC accumulation was estimated by associating, within each forest type, the age of recovering stands in 2020 derived from the TMF disturbance product with their corresponding AGC values predicted from our 2020 map. We quantified the median AGC across each age of recovering forests and fitted a Chapman-Richard model for growth (Richards, 1959) to model the AGC accumulation with increasing ages.

$$\square_{\square} = \square(1 - \square^{-\square\square})^{\square} \pm \varepsilon\,;\ \square, \square\ \square\square\square\ c > 0 \qquad (1)$$

Where $\square_{\square}$ refers to the AGC at year (*t*); $\square$ is the AGC asymptote or the AGC of the old growth forest; $\square$ is a growth-rate coefficient of $\square$ as a function of age; $\square$ is a coefficient that determines the shape of the growth curve; and ε is an error term.

For the 1-year-old recovering forests from clearing, we considered the 25th quantile of AGC to correct for potential bias on our AGC predictions for that age and ensure that the fit begins near 0 (Heinrich et al., 2021). This correction was not necessary for degraded forests as degradation still maintains some level of AGC after disturbance (Heinrich et al., 2023). After a given number of years, the AGC of recovering forests would return to amounts equivalent to undisturbed old-growth forests which will constitute the precalculated asymptote (*A*) in our equation. For each forest type, we considered the 95th quantile of AGC found within intact forests in 2020 (Potapov et al., 2017) as the respective *(A)* term in the equation.

To quantify the carbon gains of the region, we multiplied our modelled rates of annual AGC accumulation with the respective areas of forests recovering from clearing and degradation for each forest type and land use. Our estimates of annual AGC accumulation for young (≤ 20 years) recovering forests from clearing an degradation (2.09 ± 0.15 Mg C ha-1 yr-1 and 1.7 ± 0.19 Mg C ha-1 yr-1) are similar but more conservative to estimates from Heinrich et al. (2023) over Central Africa (2.51 ± 2.4 Mg C ha-1 yr-1 and 1.98 ± 0.4 Mg C ha-1 yr-1) using a space for time approach. For old growth forests which include undisturbed TMF cover and disturbed forests older than 30 years, we used the mean rate of recovering forests beyond 30 years (0.59 ± 0.42 Mg C ha-1 yr-1; Table S1) as the removal factor. This estimated carbon removal rate of old growth forests falls within the range measured from field plots in the region (0.53 - 0.79 Mg C ha-1 yr-1; Hubau et al., 2020).

Total AGC losses were derived by combining emissions from forest clearing and degradation. For clearing, we used the TMF deforestation dataset to identify the year of pixel loss from old growth and degraded forests and multiplied the total cleared area between 1990 and 2020 by their respective median AGC, assuming complete carbon loss. For degradation, we estimated losses as the difference between AGC in old growth forests and the modelled AGC during the first year following the degradation event (Heinrich et al., 2023) for each forest type and land use. These differences served as emission factors for degraded forests (see Table S2 below) and were multiplied by the degraded forest area in 2020 to quantify total AGC losses from degradation.

All these details are found in the methodological sections Quantifying carbon emissions and recovery (L432-447) and Quantifying forest carbon gains and losses (L462-479)

Table S2: Aboveground carbon accumulation rates (ΔAGC, Mg C $ha^{-1}y^{-1}$) and density (AGC, Mg C ha^{-1}) for recovering forests across land-uses and chronosequence, with 10-year intervals following the last disturbance.

Age (years)	Certified concessions		Uncertified concessions		Protected		Unprotected	
	ΔAGC	AGC	ΔAGC	AGC	ΔAGC	AGC	ΔAGC	AGC
	Forest clearing							
≤10	2.95 ±0.2	32.56 ±11.9	2.64 ±0.17	25.99 ±10.5	2.51 ±0.17	24.76 ±13.9	2.9 ±0.16	34.76 ±10.8
10-20	1.49 ±0.11	53.2 ±10.4	1.46 ±0.14	45.37 ±8.8	1.36 ±0.15	42.43 ±16.3	1.39 ±0.11	54.90 ±8.5
20-30	1.12 ±0.17	66.34 ± 8.8	1.14 ±0.18	58.49±8.9	1.06±0.2	54.34±9.9	1.03 ±0.13	67.33±7.08
>30	0.82 ±0.33	84.78 ± 16.6	0.85 ± 0.33	77.53±16.4	0.81 ±0.38	71.81±18.9	0.74±0.23	84.44±12.1
	Forest degradation							
≤10	2.5 ±0.24	77.14 ±19.7	2.58 ±0.22	66.95 ±19.8	2.49 ±0.26	60.78 ±12.7	2.48 ±0.21	63.1 ±9.1
10-20	0.84 ±0.16	91.43 ±17.7	0.92 ±0.16	82.21 ±17.5	0.89 ±0.18	75.33 ±8.4	0.87 ±0.13	77.84 ±7.1
20-30	0.55 ±0.21	98.42±10.6	0.61 ±0.21	89.92±10.4	0.59 ±0.23	82.72±11.4	0.57 ±0.17	85.21±8.6
>30	0.34±0.29	106.62±14.8	0.39±0.29	99.35±15.1	0.38±0.33	91.77±16.4	0.36±0.24	94.09±12.3

2. The Discussion section is weak and lacks strong data analysis or evidence to support the major findings of this study.

Thank you for making this point. This comment aligns with the suggestion made by the first reviewer on the discussion section (see above for more details). We have provided more details on the methodology to support the strength and relevance of our analysis and supplemented the discussion with quantitative evidence from our results and the literature to support our findings. We have also provided more arguments to connect our findings with the role of forest management in carbon mitigation in the Congo basin. We believe all these major adjustments contributed significantly to strengthening the discussion section. We therefore invite the reviewer to refer to our responses on the last 3 comments from Reviewer 1 above on the discussion section.

3. The writing of this paper has obvious errors and needs to be carefully double-checked. For example,

1) Line 27, "dominated my 98% removals from managed and intact forests"

While the '98%' estimate was not incorrect, but rather ambiguous in its meaning, the sentence has been updated for more clarity. "Old growth forests dominated the total AGC removals across land-uses (84%) with the Congo Basin rainforest functioning as a net AGC sink at -37.5 ±4.8 TgCyr-1, primarily driven by managed land-uses (99%) including logging concessions (-21.3 ±2.4 TgCyr-1) and protected areas (-15.7 ±2.2 TgCyr-1), while unmanaged areas remained nearly neutral." Line 27-31

2) Line 82 "protected unprotected areas"

Thanks for catching this typo. Now the sentence reads “We analyzed AGC distribution across the main forest types (central and coastal evergreen, semi-deciduous, peatlands and mangroves; Dinerstein et al., 2017; Réjou-Méchain et al., 2021), and land-use management regimes, including certified and uncertified logging concessions, protected and unprotected areas.” Line 83-86

3) Line 95-104 in the Results and Line 205-210 in the Discussion showed repeated writing but different or opposite conclusions.
We thank the reviewer for bringing this up. We have updated the section removing the repetition and discussed how regionally trained AGC models can improve the accuracy of AGC estimates as compared to global products. The section now reads “The uncertainty in the AGC map-based inference for carbon density and changes across forest types and land uses remain small because of our innovative integration of satellite predictors with advanced geospatial modeling techniques. This includes: 1) employing locally trained machine learning (ML) algorithms within 20 km × 20 km grid cells to interpolate canopy height metrics from GEDI; and 2) leveraging the most extensive LiDAR-derived AGC samples available over the region (~825,000 ha; Table 2) to calibrate stratified ML algorithms for each forest type and predict AGC. The localized ML approach integrates the spatial variability in the forest structure and has been demonstrated to provide better performance in predicting canopy structure at regional (Csillik et al., 2019; Sagang et al., 2024) and global (Potapov et al., 2021) scales. Upscaling AGC estimates from local to regional scales has been improved by integrating LiDAR-derived AGC samples (Réjou-Méchain et al., 2019; Sagang et al., 2020). The latter provides more comprehensive sampling and therefore a better characterization of the forest structure across the AGC gradient compared to limited field plot data. Global products either overestimated by 15% (CCI, 154.4 ± 38.7 Mg C ha-1) or underestimated by 28.9% (GEDI L4B, 95.3 ± 34 Mg C ha-1) compared to our regional mean AGC estimate (134.1 ± 35.1 Mg C ha-1). This underscores potential large uncertainties when relying on non-probabilistically sampled field inventory data or global maps to assess the contribution of the Congo Basin rainforest to global climate change mitigation efforts like the United Nations Framework Convention on Climate Change (UNFCCC), and highlights the need for locally calibrated, wall-to-wall products improved by intermediate LiDAR-derived AGC data.” Line 208-227

4) The numbering of figures and tables in the manuscript was messed up.
We thank the reviewer and agree with this remark. The table numbers mistakenly started with “Table 2” instead of “Table 1”. We have corrected this mistake and updated the reference of the table and figure numbers in the manuscript accordingly.

Minor comments:
1. What is the definition of managed forest in this study?
Managed forests in this study refers to those allocated to logging concessions for selective timber harvesting (with or without certified management plans) and protected for biodiversity conservation unlike unmanaged forests in unprotected areas which are subject to unsustainable land use. We updated the text to clarify this “This hinders our ability to assess how forest carbon stocks and fluxes vary across managed (logging concessions, protected areas) and unmanaged forests, predict their response to global changes and ultimately inform decision-making to protect and govern the Congo Basin rainforest in ways that also support economic growth.” L55-59

2. The method and process for calculating wall-to-wall canopy height are unclear.

We thank the reviewer for this remark. While we are not sure what specific part of the method was unclear for the reviewer, we have given overall updates to improve the clarity in the methodology for calculating wall-to-wall canopy height maps with their validation as detailed in section “Mapping forest structure-Canopy height”. We have developed this methodology in a previous study in the region (Sagang et al., 2024) and his has been demonstrated to provide better performances in predicting canopy structure at regional (Csillik et al., 2019; Sagang et al., 2024) and global (Potapov et al., 2021) scales.
“Satellite data from the Harmonized Landsat Sentinel-2 (HLS; Claverie et al., 2018) and ALOS PALSAR (Shimada et al., 2014) available in 2020, were used as variables to interpolate canopy relative height (RH) measurements from GEDI (Dubayah et al., 2021) into wall-to-wall canopy height maps. Optical inputs included the near-infrared (NIR), shortwave infrared (SWIR 1 & SWIR 2), and the Normalized Difference Vegetation Index (NDVI), while the horizontal (HH) and vertical (HV) polarizations were considered as predictors from radar. We retained the GEDI metrics RH50, RH75, and RH98 corresponding to the 50th, 75th, and 98th percentile of energy return height relative to the ground to capture the vertical structure of the different strata of the forest. The 25 m GEDI data available over the area between 2019 and 2020 were aggregated to a 100 m resolution using spatial averaging. We then set aside 20% of the 100 m GEDI pixels randomly selected for validation and the remaining 80% were subsequently incorporated into localized Gradient Boosted Regression Trees (GBRT; Friedman, 2002) models. GBRT models have been proven successful in predicting forest structural metrics with good performance (Colin et al., 2017; Xu et al., 2021). These models (GBRT-RH) were trained and applied within 20 × 20 km tiles with 70% overlap, ensuring that a minimum of 500 GEDI pixels were available to train the model within the current tile and randomly sampling pixels from adjacent tiles if the condition was not met (see Sagang et al., 2024 for more details). This resulted in three canopy height predictions (RH50, RH75, and RH98) for each tile for circa 2020 at 1-ha resolution. A wall-to-wall RH prediction over the Congo Basin (Figure S1) was obtained by mosaicking estimates from each tile considering the median value of overlapping pixels to minimize discontinuity effects between tiles (Csillik et al., 2019).” Line 387-407

3. The maps of the ages of recovering forests and canopy height are critical data in this study. Please add the spatial maps of these two datasets.
Thank you for these valuable suggestions. We have provided the maps in the supplementary, Figure S1 for canopy height and Figure S6 for the ages of forest recovery. See below.

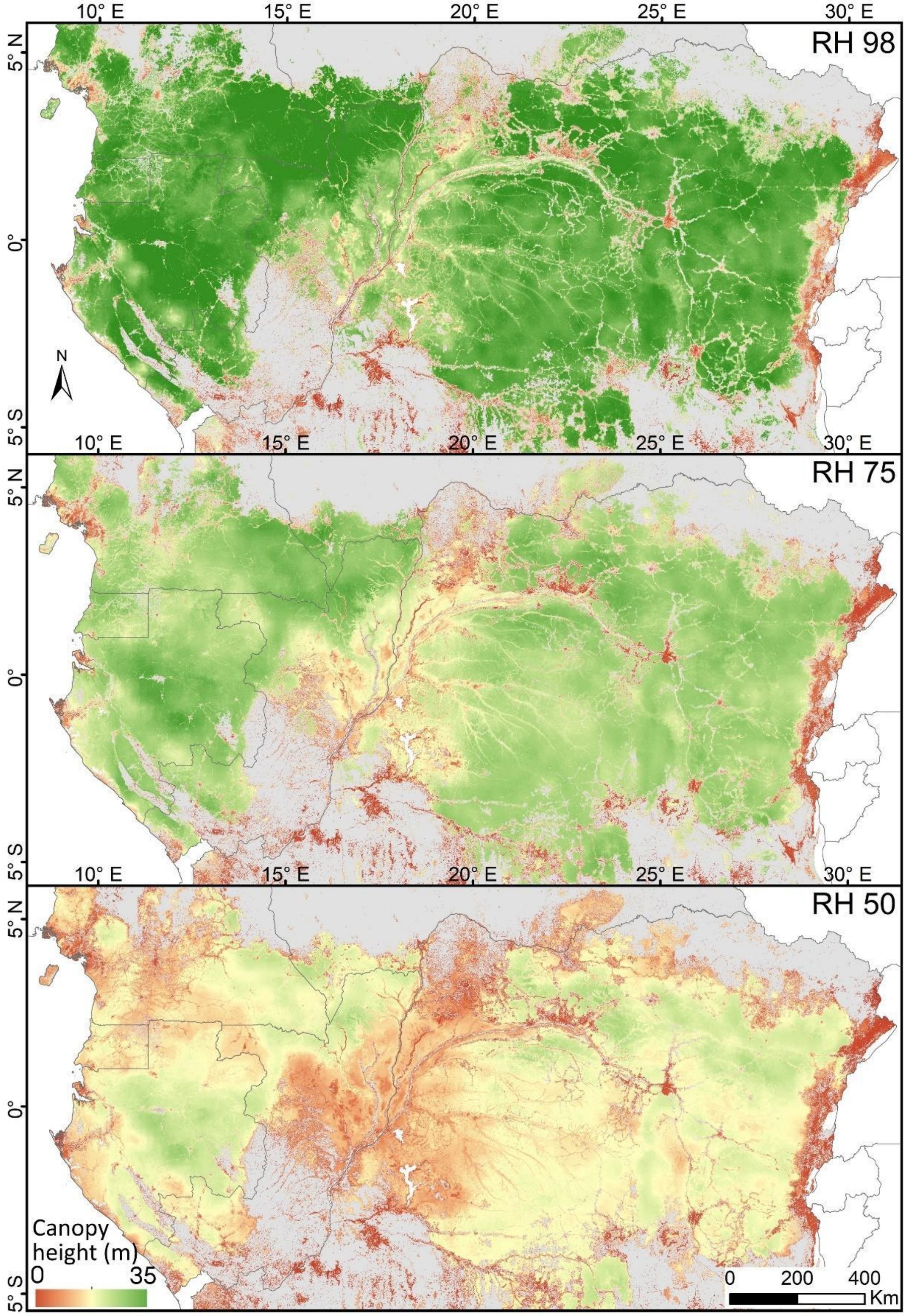

Figure S1: Wall-to-wall GEDI relative canopy height (m) predictions over the Congo basin rainforest for the year circa 2020 at 100 m spatial resolution. RH98 = top; RH75 = middle, RH50 = bottom.

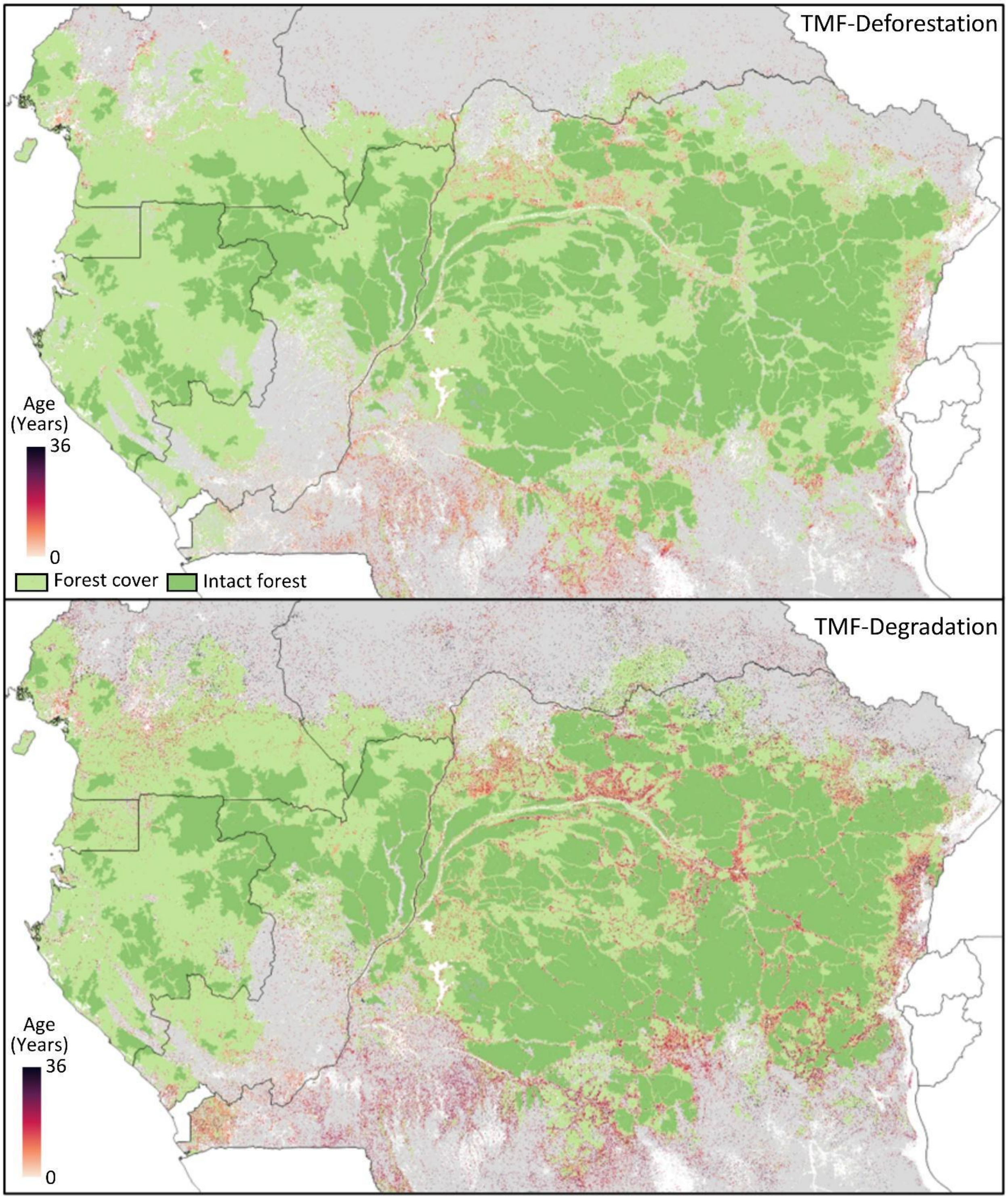

Figure S6: Age in 2020 of recovering forests from clearing (top) and degradation (bottom) as defined in the Tropical Moist Forest cover change dataset (Vancutsem et al., 2021) with intact forests in 2020 displayed as dark green.

Reviewer #2 (Remarks on code availability):
I could not open the code link.
The link has been updated. https://doi.org/10.5281/zenodo.15493192

<u>**Reviewer #3 (Remarks to the Author):**</u>

The manuscript is very well written. The topic is relevant because it supports the discussion on pros and cons of forest management with high-quality information. The applied methods are well explained and the discussion section is well structured.

I recommend the manuscript for publication.

minor edits:

l. 27: I assume 'my' should be 'by'.

Updated. Now the sentence reads "Old growth forests dominated the total AGC removals across land-uses (84%) with the Congo Basin rainforest functioning as a net AGC sink at -37.5 ±4.8 TgCyr-1, primarily driven by managed land-uses (99%) including logging concessions (-21.3 ±2.4 TgCyr-1) and protected areas (-15.7 ±2.2 TgCyr-1), while unmanaged areas remained nearly neutral." Line 27-31

l. 514: I assume the link will be provided after publication.

The link has been updated.

References: some of the cited papers do not show a publisher or a webpage. (Example Kauffman Donato: Kauffmann, J. B. & Donato, D. Protocols for the measurement, monitoring and reporting of structure, biomass and carbon stocks in mangrove forests. Working Paper 86. (CIFOR (2012)).)

Updated. "Kauffman, B., Donato, D., 2012. Protocols for the measurement, monitoring and reporting of structure, biomass and carbon stocks in mangrove forests. Center for International Forestry Research. Working Paper 86. (CIFOR (2012)).)." Line 701-703

Reviewer #3 (Remarks on code availability):

the code is not yet available.

The link has been updated. https://doi.org/10.5281/zenodo.15493192

RESPONSE TO REFEREES

We thank the reviewers for their time and the constructive feedback provided, which has significantly strengthened the manuscript. In response, we have performed an extensive revision, including comprehensive linguistic and stylistic refinements to the abstract and main text. Furthermore, we have updated all figures and labels to strictly adhere to the journal's editorial guidelines. To support transparency and reproducibility, we have meticulously referenced all datasets and included a comprehensive README.txt file alongside our code to ensure its reproducibility. We believe the revised manuscript now meets the rigorous standards of Nature Communications. We provide below our point-by-point responses.

Reviewer #1 (Remarks to the Author):

I have carefully reviewed the revised version of the manuscript and the authors' detailed responses to the previous comments. Overall, I am very satisfied with the revisions and commend the authors for their thorough and thoughtful efforts in addressing the reviewer concerns. The authors have responded comprehensively and constructively to all major and minor comments, and the manuscript is now scientifically robust, clearly presented, and suitable for publication. I have no further substantive comments and fully support its acceptance.

That said, before final publication, the manuscript would benefit from one final round of careful language polishing, particularly to ensure consistent use of verb tense throughout the Abstract and main text. At present, the Abstract occasionally mixes present and past tense within the same logical sequence (e.g., "drives," "remain," "underscore" versus "exhibited," "remained," "dominated"). Addressing these minor grammatical and stylistic issues will further improve clarity and readability.

We thank the reviewer for his constructive comments that helped to improve the robustness of the manuscript. We have implemented thorough language polishing of the manuscript, discussing the results in present tense in the abstract and addressing grammatical and stylistic issues across the current version of the manuscript.

Reviewer #1 (Remarks on code availability):

I was able to access the code repository via the provided link. The code is written in R and uses .fst files for data storage and processing, which is suitable for efficient handling of large datasets. The scripts appear to follow a logical structure for reading and processing the data.

However, the repository does not currently include a README file or equivalent documentation describing package dependencies, data structure, or instructions for running the code. As a result, while the code itself is accessible and readable for users familiar with R, its reproducibility and reusability by the broader community would be improved by the inclusion of basic documentation. I recommend that the authors add a short README outlining the required packages, input data formats, and execution workflow prior to publication.

We have provided a README.txt file describing package dependencies, data structure, and instructions for running the code to reproduce all the results presented in the manuscript. See files here https://doi.org/10.5281/zenodo.18930528

Reviewer #2 (Remarks to the Author):

The authors have addressed my concern. I am happy to accept this paper for publication.

We thank the reviewer for his constructive recommendations that shaped the improvement of this manuscript.
